# Fetal growth in environmental epidemiology: mechanisms, limitations, and a review of associations with biomarkers of non-persistent chemical exposures during pregnancy

**DOI:** 10.1186/s12940-019-0480-8

**Published:** 2019-05-08

**Authors:** Elizabeth M. Kamai, Thomas F. McElrath, Kelly K. Ferguson

**Affiliations:** 1 Department of Epidemiology, Gillings School of Global Public Health, University of North Carolina at Chapel Hill, 135 Dauer Drive, 2101 McGavran-Greenberg Hall, CB #7435, Chapel Hill, NC 27599 USA; 2 Division of Maternal-Fetal Medicine, Department of Obstetrics and Gynecology, Brigham and Women’s Hospital, Harvard Medical School, 75 Francis Street, Boston, MA 02115 USA; 3 Epidemiology Branch, Division of Intramural Research, National Institute of Environmental Health Sciences, 111 TW Alexander Drive, Research Triangle Park, NC 27709 USA

**Keywords:** Biomarkers, fetal growth, non-persistent, phthalates, phenols, pesticides, birth weight

## Abstract

**Background:**

Non-persistent chemicals, such as phthalates, environmental phenols, organophosphate pesticides, and others, are challenging to study because of their ubiquity in the environment, diverse exposure routes, and high temporal variability of biomarkers. Nonetheless, there is interest in understanding how gestational exposure to these chemicals may affect fetal growth, as perturbations to normal fetal growth are related to a plethora of adverse health outcomes in childhood and adulthood.

**Methods:**

The purpose of this review is to describe the state of the science on this topic. We searched PubMed for studies that included both 1) biomarkers of non-persistent chemicals collected during pregnancy and 2) fetal growth outcomes measured at birth (e.g., birth weight) or by ultrasound *in utero* (e.g., estimated fetal weight).

**Results:**

The bulk of the literature we found uses biomarkers measured at a single time point in pregnancy and birth weight as the primary measure of fetal growth. There is a small, but growing, body of research that uses ultrasound measures to assess fetal growth during pregnancy. In addition to summarizing the findings of the publications we identified, we describe inconsistencies in methodology, areas for improvement, and gaps in existing knowledge that can be targeted for improvement in future work. This literature is characterized by variability in methodology, likely contributing to the inconsistency of results reported. We further discuss maternal, placental, and fetal pathways by which these classes of chemicals may affect fetal growth.

**Conclusions:**

To improve understanding of how everyday chemical exposures affect fetal growth, and ultimately lifelong health outcomes, mechanisms of toxicant action should be considered alongside improved study designs for future hypothesis-driven research.

**Electronic supplementary material:**

The online version of this article (10.1186/s12940-019-0480-8) contains supplementary material, which is available to authorized users.

## Background

Birth weight is among the most commonly studied health outcomes in environmental epidemiology. It is readily acquired through birth records, has reliable recall, and is less subject to measurement error compared to other pregnancy outcomes (e.g., gestational age at delivery). Extreme low or high birth weight is a well-known risk factor for neonatal mortality and various morbidities in infancy, adolescence, and adulthood [1–6].

An alternative approach to examining fetal growth involves collecting ultrasound parameters of fetal size at multiple time points during pregnancy in conjunction with metrics at delivery. Using repeated ultrasound measures to assess growth reduces measurement error and allows for the assessment of growth over time. This approach may also enable identification of windows of gestation where growth is more sensitive to environmental perturbations and, with the availability of parameters beyond weight to estimate size, individual compartments that are particularly affected.

Recent reviews have highlighted the associations between fetal growth and environmental exposures such as air pollutants and persistent organics pollutants [7, 8]. However, associations with non-persistent environmental contaminants have not been specifically examined, and are of particular interest due to their ubiquity and potential for endocrine disruption [9–12]. Additionally, these chemicals, such as phthalates, environmental phenols, parabens, non-persistent pesticides, and organophosphate ester flame retardants, add complexity to the study of fetal growth due to their diverse exposure routes and the short half-lives of their available biomarkers [9, 13].

Herein we describe some potential etiologic mechanisms of environmental toxicant action on fetal growth. We then provide a comprehensive review of the studies that have examined these non-persistent chemical exposures during pregnancy in relation to the fetal growth measures described above. We summarize the available studies, followed by a discussion and interpretation of inconsistencies in methodology and synthesis of gaps in existing knowledge that can be targeted for improvement in future work.

## Potential Etiologic Mechanisms

**Fig. 1:**
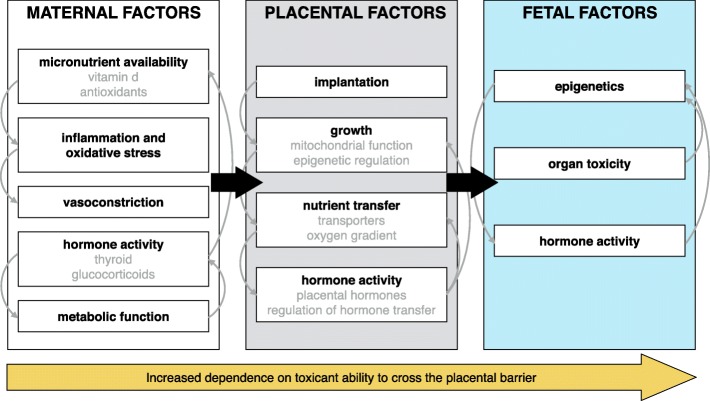
Maternal, placental, and fetal factors in fetal growth that may be sensitive targets of environmental chemical exposures

The mechanisms underlying associations between non-persistent environmental contaminant exposures and fetal growth restriction are poorly understood. However, there is strong biologic plausibility and animal evidence for mechanisms that could drive these perturbations. Here we summarize some of the known maternal, placental, and fetal factors associated with reduced fetal growth and offer some examples of how non-persistent environmental contaminants could act through these pathways (Fig. 1).

### Maternal factors

Maternal environment is a major factor in fetal growth and may be more important than genetics. This is exemplified in a study of pregnancies with ovum donation in which the authors observed correlations between birth weight of the mother, but not the ovum donor, and birth weight of the newborn [14]. The following characteristics of the maternal environment may be particularly important for fetal growth, and at the same time may be sensitive to environmental chemical exposures. Most of these factors likely act by influencing the placental implantation, growth, and nutrient transfer, or by causing changes in the fetus that influence growth.

Maternal nutrient intake is one of the strongest risk factors for fetal growth restriction [15]. Extreme maternal nutrient deprivation, as in times of famine, is the best example [16]. Effects of modest changes in micronutrient intake are more ambiguous, but there is some epidemiologic evidence for association. Decreased maternal serum concentrations of 25-hydroxyvitamin D (25OHD) during pregnancy have been associated with size for gestational age [17], and reduced bone growth [18]. Some phthalate metabolites and bisphenol A (BPA) have been associated with circulating 25OHD levels in adults, including in pregnant women [19, 20]. These compounds may perturb the normal metabolism of the compound, for example by altering the activity of cytochrome P450 enzymes, or directly interfere with the vitamin D endocrine axis [21, 22]. Other micronutrients, such as antioxidants (e.g., Vitamins C or E), have minimal evidence for an effect on fetal growth [23], but many non-persistent compounds have been associated with increased maternal oxidative stress [24, 25].

There is strong evidence for an association between maternal oxidative stress and inflammation and fetal growth restriction. Residing in areas of high altitude, which leads to hypoxia and oxidative stress [26], is consistently associated with reduced fetal growth, although the consequences of this association are unclear [27, 28]. Maternal inflammation, tightly connected to oxidative stress, also causes growth restriction (e.g., in examples of maternal infection [29, 30]). Epidemiologic studies examining circulating biomarkers of inflammation and oxidative stress also support a relationship between these factors and reduced fetal growth [31, 32]. These effects are likely mediated through poor invasion of the trophoblast in placental development, as well as altered spiral arteriole remodeling [33, 34]. Phthalates [25], environmental phenols [35], and non-persistent pesticides [36] have been suggestively associated with oxidative stress and inflammation in animal as well as human studies, making this a plausible mechanism underlying exposure and fetal growth restriction associations.

Independently or in connection with these pathways, maternal vasoconstriction and elevated blood pressure are important risk factors for fetal growth restriction that can be influenced by the environment. By way of impairing placentation and reducing nutrient delivery to the fetus, vascular disease is considered one of the most common causes of growth restriction [37]. Cigarette smoke, which has been associated with reduced birth weight by up to 150 grams [38], likely acts at least in part through vasoconstrictive effects of nicotine [39]. Urinary phthalate metabolites and BPA have been associated with elevated blood pressure, although these studies have been primarily cross-sectional [40–42].

Probably the most plausible mechanism for associations between these compounds and growth restriction is through endocrine disruption pathways. Phthalates, environmental phenols, and many pesticides fall under the classification of Endocrine Disrupting Compounds (EDCs) because of their ability to interfere with hormones [43]. Furthermore, many of the associations between these compounds and adverse health outcomes demonstrate sex-specific effects [44, 45]. Clinical as well as sub-clinical changes in maternal hormone levels in pregnancy play a key role in development of the placenta and fetus [46]. Thyroid hormones are essential for fetal growth and other endocrine regulation [47], and derive from the mother primarily for the first half of pregnancy [48]. Estrogens, androgens, glucocorticoids, insulin, gonadotropins, and growth factors (e.g., insulin-like growth factor 2, or IGF-2) also play important roles in regulating nutrient delivery to the fetus as well as organ maturation [49–51]. Thyroid hormones and neuroendocrine systems in particular may be important targets of environmental contaminants [12]; other pathways have been less explored but deserve more attention, especially in the context of pregnancy. Furthermore, the potential involvement of these pathways in the associations between maternal exposure and fetal growth make it imperative that associations in epidemiologic studies be carefully examined for evidence of effect measure modification by infant sex.

Lastly, and largely entangled with endocrine factors, maternal metabolic function plays a major role in regulating fetal growth. Hyperglycemia, adiposity, and type 2 as well as gestational diabetes are associated with increased fetal growth [52, 53]. Many of the compounds described in this review are suspected obesogens with the capacity to dysregulate glucose homeostasis, primarily through interacting with peroxisome proliferator activated receptors (PPARs) [53]. Thus, researchers should be attentive to the potential for *overgrowth* of the fetus in response to chemical exposures as well.

### Placental factors

Changes in the maternal environment can have a major influence on implantation, growth, nutrient transfer, and hormonal activity of the placenta. For example, oxidative stress early in pregnancy may interfere with normal trophoblast invasion and widening of spiral arterioles, leading to insufficient vascularization of the placenta [54]. Additionally, if these chemicals cross the placental barrier, as most of them do, they can influence these processes in a more direct manner. This is extremely important as low nutrient supply to the fetus is the number one cause of fetal growth restriction, and the placenta is the rate-limiting-factor in nutrient transfer.

A major target of environmental exposures in the placenta could be epigenetic factors, including methylation, histone modifications, and miRNA activity, which affect transcription and expression of genes. IGF-2 expression in the placenta is particularly important for its growth and for regulation of nutrient delivery to the fetus [55]. Environmental factors may interfere with IGF-2 imprinting and consequently change the normal programing under which the placenta and fetus develop [56]. While there is stronger evidence for dietary factors such as folic acid and modifications in methylation of imprinted genes [57], there is also some evidence for an association with chemicals like BPA and phthalates in animal as well as human studies [58, 59]. In addition to methylation patterns, these compounds may also influence other epigenetic factors such as histone modifications or transcription factors like miRNAs, although research in this direction is more recent and limited [60, 61].

Placental mitochondria are another potential target of environmental toxicants [62]. Mitochondrial function in the placenta is of great interest because of the high metabolic activity of this organ and the connection between mitochondrial production of, as well as sensitivity to, oxidative stress. Oxidative stress can paradoxically lead to increases as well as decreases in mitochondrial DNA content, as compared to nuclear DNA content, depending on the magnitude of the insult and timing [62]. Thus, a higher proportion of mitochondrial DNA in the placenta may reflect damage and either appropriate and effective compensation, or inefficient compensation resulting in poorer respiration of each unit. At the same time, a decreased proportion of mitochondrial DNA could also be adverse, reflecting chronic oxidative stress and inability to compensate [62]. Both lower as well as higher mitochondrial DNA content (relative to nuclear DNA content) has been observed in placentas from intrauterine growth restriction (IUGR) pregnancies compared to normal pregnancies, and both can be justified as contributing to growth restriction [63, 64]. In addition, several studies have observed associations between air pollutants or persistent EDCs and placental mitochondrial DNA content and have interpreted both directions as having potentially negative consequences for pregnancy [65–67]. While this target may be important for environmental exposures, additional basic science to understand the meaning behind the biomarkers of placental mitochondrial function is necessary.

Nutrient transporters in the placenta may also be sensitive to environmental toxicants that make their way into the tissue, which occurs commonly for the chemicals discussed in this review. This could occur through direct interaction with activate transporters, as with the observed disruption of the amino acid transporter systems by nicotine and cocaine [68, 69], or by interference with hormonal systems that regulate transport [70]. In one mouse study, altered nutrient transporter gene expression was observed in association with mono-2-ethylhexyl phthalate administration [71] .However, to our knowledge, these relationships have not been examined in studies using human placentas.

Lastly, the placenta is an endocrine organ itself and generates hormones in pregnancy that play a major regulatory role in maintaining pregnancy and in the growth of the fetus. Key players include estrogen and progesterone, placental lactogen, placental growth hormone, and placental corticotropin-releasing hormone [70]. In *in vitro* studies some toxicants have demonstrated ability to inhibit secretion of hormones from placenta-specific cells [72]. However, less evidence exists in human studies. Beyond changes in hormone production, there are also enzymes secreted by the placenta that protect against effects of maternally circulated hormones. The best example is 11ß-hydroxysteroid dehydrogenase 2 (11ß-HSD2), which converts cortisol from the mother, thought to inhibit growth, into the inactive cortisone, thus protecting the fetus. Reductions in placental 11ß-HSD2 are strongly associated with growth restriction [73]. A number of environmental contaminants, particularly phthalates and carbamate pesticides, have been shown to reduce 11ß-HSD2 activity *in vitro*, but exploration of this mechanism in human populations remains to be seen [74, 75].

### Fetal factors

Congenital anomalies in the fetus, such as trisomy, are associated with fetal growth restriction, although whether they are a cause or consequence is not clear [76]. Nevertheless, they may stem from the same underlying factor, genetic or environmental. Environmental chemical exposures also have been associated with human congenital malformations [77]. Once chemicals pass through the placental barrier, the fetus may be at greater risk to their toxicity because of its rapid development and the reduced capacity for detoxification [78]. Damage to the thyroid gland, immunotoxicity, and neurotoxicity may be ready targets that could influence the ability of the fetus to grow normally.

As with the placenta, genes regulated by epigenetic markers in the fetus are important for normal growth and may be sensitive to environmental exposures. (In fact, fetal epigenetic changes have been studied much more in the context of environmental exposure than placental changes.) IGF-2 in the fetus influences the nutrient demand, which is one of the most important factors for fetal development, but epigenetic modifications in other imprinted and non-imprinted genes may be influential as well.

Finally, as pregnancy progresses, and the fetus begins to produce hormones on its own, the endocrine disrupting effects of these compounds that have been noted in the mother may occur in the fetus as well. In fact, the fetus may be even more sensitive to toxic effects of these compounds as mentioned above. Some studies have demonstrated associations between *in utero* exposure to non-persistent chemical exposures in pregnancy and changes in cord blood hormone levels, which are thought to reflect fetal effects. For example, di (2-ethylhexyl) phthalate (DEHP) metabolites in urine have been associated with decreased fetal testosterone levels measured in cord blood in females [79], and a decrease in insulin-like factors and other hormones in males [80]. Likewise, maternal phthalate exposure in pregnancy has been associated with changes in cord blood thyroid hormone concentrations [81]. These hormonal changes could thus influence normal growth of the fetus as well.

## Methods

We searched PubMed for studies published in English available online through June 2018 using combinations of key words for non-persistent environmental exposures and fetal growth outcomes (see Additional file 1 “Keywords for literature review”). More than 3000 results were produced. Titles and abstracts were reviewed by EK, and relevant articles were examined using the following additional criteria. We only included studies that measured one or more biomarkers of exposure, and excluded studies based on self-report or occupational exposures assigned via job exposure matrix. We further excluded studies that did not measure the chemical of interest (or its metabolite), such as those that only used biomarkers of acetylcholinesterase inhibition as measures of pesticide exposures. We included only studies that reported physical size as growth outcomes (e.g., crown-rump length, femur length, biparietal distance). We excluded studies based solely on biomarkers of growth or obesity like leptin, total cholesterol, or triglycerides. Furthermore, although reported in some studies included in our review, we did not include anogenital distance as an outcome of fetal growth as this is a more targeted marker of androgen action and sexual dimorphism rather than overall physical development [82].

We organized results by three primary chemical groups: A) phthalates, B) environmental phenols and other non-persistent consumer product chemicals, and C) non-persistent pesticides. We further considered three sets of criteria: 1) whether the chemical or metabolite of interest was measured in urine or in a different matrix; 2) whether the study measured exposure at multiple time points; and 3) whether the study examined fetal growth measured *in utero* (e.g., ultrasound measures of fetal size or diagnosis of IUGR by ultrasound) or size measured at birth (e.g., birth weight or birth length). Among studies that used ultrasound measures, we further distinguish between clinical diagnoses of IUGR and ultrasound measures collected for research purposes. We included studies that defined IUGR as estimated fetal weight in the lowest 10^th^ percentile for gestational age. In our presentation of results, we focused on findings that were statistically significant at an alpha level of 0.05. When results were analyzed both with and without adjustment for gestational age, we presented results without adjustment in tables, as gestational age may be a mediator along the causal path between toxicant exposure and fetal growth [83]. We present results stratified by sex or restricted to a single sex, as the effects of prenatal exposure to some non-persistent environmental chemicals may differ by fetal sex. For studies of organophosphate pesticides, we additionally present results stratified by *PON1* genotype and status. Finally, because urine is the preferred matrix for measuring non-persistent organic pollutants [13, 84], we focused on these results in the primary tables but mentioned studies assessing exposure in other biological specimens in the text and supplemental tables.

## Results of review

### Phthalates

Phthalates are a group of chemicals typically used as plasticizers in a wide variety of industrial and consumer products, including polyvinyl chloride products, medical devices, food packaging, toys, and personal care products [85, 86]. Because of the pervasive use of these chemicals and the ease with which they are leached from products into the environment, human exposure to phthalates is nearly ubiquitous in the United States (U.S.) and Europe [87, 88]. Phthalates are often categorized into two groups based on molecular weight: low molecular weight phthalates are <250 g/mol and include dimethyl phthalate, diethyl phthalate, di-*n*-butyl phthalate, and diisobutyl phthalate; high molecular weight phthalates include butylbenzyl phthalate, di (2-ethylhexyl) phthalate (DEHP), di-*n-*octyl phthalate, diisononyl phthalate [89, 90]**.** This classification reflects both structural similarity and similar routes of exposure, as low molecular weight phthalates are often found in personal care and hygiene products, while high molecular weight phthalates are commonly used as plasticizers in polyvinyl chloride (PVC) materials, tubing, medical devices, and food packaging [85, 87]**.** Humans are exposed to some phthalates such as DEHP, and increasingly, diisononyl phthalate, through contaminated food and drinking water [90, 91]. Exposure to other high production volume phthalates – including diethyl phthalate (found primarily in fragrances), dibutyl phthalate, di-*n*-butyl phthalate, diiosbutyl phthalate, and butylbenzyl phthalate – is likely from the use of consumer goods and personal care products [92, 93]. Metabolites of phthalates are excreted in urine within a matter of hours, and exposure (and the amount excreted) can vary within a single day [94].

#### Phthalates and fetal growth outcomes measured at birth

We identified 19 studies conducted in China, France, Spain, Poland, Japan, Taiwan, and the U.S. that examined associations between maternal urinary phthalate metabolites and at least one growth outcome measured at birth (Table 1). The studies varied by sample size (from fewer than 100 infants to more than 3000), timing of exposure measurement (urine collected preconception [98, 109], a variety of time points during pregnancy [89, 95–97, 99–101, 103–112], or at delivery [106, 108, 113]), which phthalate metabolites were measured, outcome assessment, covariates included in multivariable models, statistical methodology, and associations reported. Most studies included multiparous and nulliparous women, all singleton, term and preterm births, and accounted for gestational age by adjustment or standardization (with some exceptions [98, 104, 105, 107, 109, 112]).

**Table 1:** Studies of maternal prenatal urinary phthalate metabolites and fetal growth outcomes measured at birth

Reference	Country, years	N	Urinary phthalate metabolites	Timing of urine collection	Birth size outcome(s)	Notes^1^	All^2^	Girls^2^	Boys^2^
Wolff et al. 2008 [89]	USA 1998-2002	404	∑LMW: MMP, MEP, MBP, MiBP ∑HMW: MBzP, MCPP, MECPP, MEHHP, MEOHP, MEHP ∑DEHP: MECPP, MEHHP, MEOHP, MEHP	3^rd^ trimester	BW BL HC	primiparas only	+ BL (MBzP) + HC (MEP, ∑LMW)		
Suzuki et al. 2010 [95]	Japan 2005-2008	149	MMP, MEP, MnBP, MBzP, MEHP, MEHHP, MEOHP, MiNP, MnOP	29 weeks	BW BL HC		NS		
Philippat et al. 2012 [96]	France 2003-2006	287	∑LMW: MEP, MBP, MiBP ∑HMW: MBzP, MCPP, MCOP, MCNP, MEHP, MEHHP, MEOHP, MECPP ∑DEHP: MEHP, MEHHP, MEOHP, MECPP	22 weeks	BW BL HC				- BW (MCPP, MECPP) - BL (MCPP)
Zhao et al. 2014 [97]	China 2012-2013	126	MBP, MMP ∑DEHP: MEHP, MEHHP, MEOHP	3^rd^ trimester	BW BL		- BW (MEHHP, MEOHP)	NS	- BW (MEHHP, MEOHP)
Smarr et al. 2015 [98]	USA 2005-2009	233	MMP, MEP, MBP, MiBP, MBzP, MECPP, MCMHP, MEOHP, MEHHP, MEHP, MCPP, MCHP, MiNP, MnOP	pre-conception	BW BL HC	not adjusted for GA	- BW (MMP, MEP, MCMHP, MOP) - BL (MMP) - HC (MEP, MEOHP, MECPP)		
Botton et al. 2016 [99]	France 2003-2006	520	∑LMW: MEP, MBP, MiBP ∑HMW: MBzP, MCOP, MCPP, MCNP, MECPP, MEHHP, MEOHP, MEHP ∑DEHP: MECPP, MEHHP, MEOHP, MEHP	26 weeks	BW BL HC				+ BL (MCNP)
Casas et al. 2016 [100]	Spain 2004-2006	390	MBzP ∑LMW: MEP, MiBP, MnBP ∑DEHP: MEHP, MEHHP, MEOHP, MECPP	12, 32 weeks*	BW BL HC		NS	+ BW (MiBP)	+ BW (MBzP, MnBP)
Ferguson et al. 2016 [101, 102]	USA 2006-2008	482	MEP, MBP, MiBP, MBzP, MCPP ∑DEHP: MECPP, MEHHP, MEOHP, MEHP	10, 18, 26, 35 weeks*	BW		NS	NS	NS
Polanska et al. 2016 [103]	Poland 2007	165	∑LMW: MEP, MiBP, MBzP, MnBP, OH-MnBP ∑HMW: MEHP, MEHHP, MEOHP, MHiNP, MOiNP, MOP ∑DEHP: MEHP, MEHHP, MEOHP ∑DBP: MnBP, OH-MnBP ∑DiNP: MHiNP, MOiNP	3^rd^ trimester	BW BL HC		NS		
Sathyanarayana et al. 2016 [104]	USA 2010-2012	674	MEP, MBzP, MBP, MCPP, MCOP ∑DEHP: MEHP, MEHHP, MEOHP, MECPP	1^st^ trimester	BW	not adjusted for GA		+ BW (MEHHP)	NS
						<37 weeks only; not adjusted for GA		+ BW (MEHP, MEHHP, MEOHP, MECPP, ∑DEHP)	NS
						≥37 weeks only; not adjusted for GA		NS	+ BW (MCOP)
Shoaff et al. 2016 [105]	USA 2003-2006	368	∑DEHP: MEHHP, MEHP, MEOHP, MECPP	16, 26 weeks*	BW BL HC	not adjusted for GA	NS		
Watkins et al. 2016 [106]	USA 2009-2012	68	MEP, MBzP, MCPP ∑DBP: MnBP, MiBP ∑DEHP: MEHP, MEHHP, MEOHP, MECPP	1^st^ trimester	BW BL HC			- BW (∑DBP) + BL (MCPP)	NS
				delivery				+ BW (MCPP) + BL (MBzP, ∑DBP) + HC (MCPP)	+ BW (∑DBP) + BL (MCPP)
				1^st^ trimester, delivery*				NS	+ BW (∑DBP) + HC (∑DBP)
Gao et al. 2017 [107]	China 2013-2014	3103	MMP, MEP, MBP, MBzP ∑DEHP: MEHP, MEHHP, MEOHP	10 weeks	BW BL HC	not adjusted for GA	- BW (MBzP)		
Huang et al. 2017 [108]	Taiwan 2010	162	∑Phthalates: MMP, MEP, MiBP, MnBP, MBzP, MEHP, MEOHP, MEHHP, MECPP	11 weeks	BW BL HC		NS		
				26 weeks			- BL (MMP, ∑Phthalates) - HC (∑Phthalates)		
				delivery			- HC (∑Phthalates)		
Messerlian et al. 2017 [109]	USA 2005-2016	364	MEP, MCPP, MCOP, MCNP ∑DEHP: MEHP, MEHHP, MEOHP, MECPP	pre-conception	BW	non-IVF births; not adjusted for GA	- BW (MCNP)		
				6, 21, 35 weeks*			+ BW (MBP, MiBP, MBzP)		
Woods et al. 2017 [110]	USA 2003-2006	272	MBP, MiBP, MEP, MBzP, MCPP, MEHP, MEHHP, MEOHP	16, 27 weeks*	BW		NS		
Chiu et al. 2018 [111]	USA 2015-2016	300	MEP, MiBP, MnBP, MBzP, MEHP, MEHHP, MEOHP, MECPP	1^st^, 2^nd^, 3^rd^ trimesters*	BW		NS		
Zhang et al. 2018 [112]	China 2013-2014	3103	MMP, MEP, MBP, MBzP ∑DEHP: MEHP, MEHHP, MEOHP	1^st^, 2^nd^, 3^rd^ trimesters*	BW	not adjusted for GA	- BW (MMP, MEP)	- BL (MMP, MEP)	NS
Zhu et al. 2018 [113]	China 2014	1002	∑LMW: MMP, MEP, MiBP, MnBP ∑DEHP: MECPP, MEHHP, MEOHP	delivery	BW BL			NS	+ BW (∑DEHP, MECPP, MEOHP)

^1^Unless otherwise specified, studies included term, preterm, parous, and nulliparous births, and models of birth outcomes adjusted for or standardized to gestational age

^2^Blank cells indicate associations were not examined; NS indicates that associations within that category were examined but not statistically significant at *p*<0.05

*Average of two or more urine samples

Abbreviations. BL, birth length; BW, birth weight; GA, gestational age; HC, head circumference; IVF, in vitro fertilization; MBP, monobutyl phthalate; MBzP, monobenzyl phthalate; MCHP, monocyclohexyl phthalate; MCOP, monocarboxy-isooctyl phthalate; MCNP, monocarboxy-isononyl phthalate; MCPP, mono-3-carboxypropyl phthalate; MCMHP, mono-[(2-carboxymethyl) hexyl] phthalate; MEP, monoethyl phthalate; MECPP, mono-2-ethyl-5-carboxypentyl phthalate; MEHHP, mono-2-ethyl-5-hydroxyhexyl phthalate; MEHP, mono-2-ethylhexyl phthalate; MEOHP, mono-2-ethyl-5-oxohexylphthalate; MHiNP, mono-hydroxy-iso-nonyl phthalate; MiBP, monoisobutyl phthalate; MiNP, mono-*iso*-nonyl phthalate; MMP, monomethyl phthalate; MnBP, mono-*n*-butyl phthalate; MnOP, mono-*n*-octyl phthalate; MOP, monooctyl phthalate; MOiNP, mono-oxo-iso-nonyl phthalate; OH-MnBP, 3OH-mono-n-butyl phthalate; ∑DBP, molar sum of the dibutyl phthalate metabolites that follow; ∑DEHP, molar sum of the di-2-ethylhexyl phthalate metabolites that follow; ∑DiNP, molar sum of the di-iso-nonyl phthalate metabolites that follow; ∑HMW, molar sum of the high molecular weight phthalate metabolites that follow; ∑LMW, molar sum of the low molecular weight phthalate metabolites that follow

Positive, negative, and null associations with size at birth were reported (Table 1). Several found no statistically significant associations between any urinary phthalate metabolites and any birth size outcome [95, 101, 103, 105, 110, 111]. Seven studies reported at least one positive association between prenatal phthalate exposure and birth weight [100, 104, 106, 109, 113], length [89, 99, 106], or head circumference [89, 106], or while eight studies found at least one inverse association with birth weight [96–98, 106, 107, 109, 112], length [96, 98, 108, 112], or head circumference [98, 108]. There were no notable patterns by phthalate metabolite or molecular weight, timing of exposure assessment, or outcome measured.

Less than half of the studies we identified modeled associations stratified by or restricted to a single infant sex, and these results were not consistent. Of these studies, seven stratified cohorts by infant sex [97, 100, 101, 104, 106, 112, 113], five of which reported formal statistical analysis of effect measure modification by infant sex by testing either interaction terms [100, 106, 112, 113] or the difference in coefficient estimates [101]. Two cohorts in France were restricted to male infants [96, 99]. Although two studies reported some inverse associations between some high molecular weight phthalate metabolites and birth weight or length in boys [96, 97], five others found positive associations between both low and high molecular weight urinary phthalate metabolites measured at different time points in pregnancy and birth size in boys [99, 100, 104, 106, 113]. Among girls, concentrations of high molecular weight phthalates generally had null [97, 102, 113] or positive associations with birth size [100, 104, 106]. Four studies found no statistically significant associations between prenatal urinary phthalate metabolite concentrations and birth size in models restricted to boys [102, 112] or to girls [97, 102, 113].

Notably, several studies utilized urinary phthalate metabolites measured at multiple time points in pregnancy [100, 101, 105, 106, 109–112]. Four averaged the concentrations of phthalates in spot urine samples collected at two time points in early and in mid-late pregnancy or at delivery to produce a single exposure estimate [100, 105, 106, 110]. Three measured urinary metabolites at three time points [109, 111, 112], and one examined phthalate measures collected at up to four times in pregnancy [101]. Generally, studies that combined repeated measures of phthalate concentrations were not statistically significantly associated with birth size outcomes [101, 105, 110, 111]. However, there were some exceptions. A study of 3100 births in China found that low molecular weight phthalate metabolites monomethyl phthalate (MMP) and monoethyl phthalate (MEP) were associated with reduced birth weight in the overall cohort and with birth length in girls [112]. On the other hand, monobenzyl phthalate (MBzP) was positively associated with birth weight in two studies [100, 109], and metabolites of dibutyl phthalate (DBP) were positively associated with birth weight in models restricted to boys [100, 106], restricted to girls [100], and overall [109].

We additionally identified 12 studies that examined prenatal exposure to phthalates using an alternative medium to maternal urine, with mixed results (Additional file 2: Table S1). Most of these studies measured phthalates in umbilical cord serum [114–120], two studies examined phthalate concentrations in maternal blood or serum collected during pregnancy [121, 122], two measured phthalates in meconium [120, 123], one utilized amniotic fluid measures [124], and one measured phthalate levels in newborns’ urine [125]. However, the preferred matrix for measuring human exposure to phthalates is urine. Phthalate levels measured in other matrices are orders of magnitude lower than levels in urine and more prone to error from contamination [13]. Results from these studies are thus not directly comparable to those that used measures phthalate exposure in maternal urine.

#### Phthalates and fetal growth outcomes measured during gestation

We identified five studies that combined *in utero* with delivery measurements to assess fetal growth (Table 2). These studies varied by size (from 119 to 520 infants), timing and number of urine samples collected, phthalate metabolites measured, outcomes assessed, covariates included in multivariable models, statistical methodology, and associations reported.

**Table 2:** Studies of maternal prenatal urinary phthalate metabolites and fetal growth outcomes measured *in utero*

Reference	Country, years	N	Urinary phthalate metabolites	Timing of urine collection	*in utero* outcome(s)	Timing of outcome assessment	Notes^1^	All^2^	Girls^2^	Boys^2^
Zhao et al. 2014 [97]	China 2012-2013	126	MBP, MMP ∑DEHP: MEHP, MEHHP, MEOHP	3^rd^ trimester	IUGR	3^rd^ trimester		+ IUGR (MEHHP)		
Zhao et al. 2015 [126]	China 2011-2013	119	MBP, MMP ∑DEHP: MEHP, MEHHP, MEOHP	3^rd^ trimester	Growth restricted (IUGR or low birth weight)	not specified		+ Growth restricted (MEHP, MEHHP, MEOHP, ∑DEHP)		
							<37 weeks only	NS		
							≥37 weeks only	+ Growth restricted (MEHHP, MEOHP, ∑DEHP)		
Botton et al. 2016 [99]	France 2003-2006	520	∑LMW: MEP, MBP, MiBP ∑HMW: MBzP, MCOP, MCPP, MCNP, MECPP, MEHHP, MEOHP, MEHP ∑DEHP: MECPP, MEHHP, MEOHP, MEHP	26 weeks	EFW AC BPD HC FL	12 weeks				- BPD (MBzP, ∑HMW, MECPP, MEHHP, MEOHP, ∑DEHP)
						22 weeks				- EFW (MBzP, MCNP) - BPD (MBzP, MCPP, ∑HMW, MECPP, MEHHP, MEOHP, MEHP, ∑DEHP) + FL (MCNP)
						32 weeks				- BPD (MCPP, ∑HMW, MECPP, MEHHP, MEOHP, MEHP, ∑DEHP) + FL (MCNP)
						overall pregnancy				- EFW (∑HMW, MECPP, MEHHP) - BPD (MBzP, ∑HMW, MECPP, MEOHP, ∑DEHP) + FL (MCNP)
Casas et al. 2016 [100]	Spain 2004-2006	390	MBzP ∑LMW: MEP, MiBP, MnBP; ∑DEHP: MEHP, MEHHP, MEOHP, MECPP	12, 32 weeks**	EFW AC BPD HC FL	12 weeks	not adjusted for GA	NS	NS	NS
						20 weeks		- HC (MnBP)	NS	NS
						34 weeks		+ FL (MBzP)	+ FL (MBzP)	NS
						growth rate 12-20 weeks		- EFW (MnBP) - HC (MnBP)	- HC (MnBP)	- HC (MnBP)
						growth rate 20-34 weeks		+ FL (MBzP)	+ FL (MBzP)	+ EFW (MnBP) + AC (MnBP)
Ferguson et al. 2016 [101, 102]	USA 2006-2008	482	MEP, MBP, MiBP, MBzP, MCPP ∑DEHP: MECPP, MEHHP, MEOHP, MEHP	10, 18, 26, 35 weeks**	EFW AC HC FL	26, 35, 38 weeks		- EFW (MEOHP, MECPP, ∑DEHP) - AC (MECPP, ∑DEHP) - HC (MECPP, MBzP) - FL (MECPP, ∑DEHP)	- EFW (MECPP, ∑DEHP) - HC (MEP)	- AC (∑DEHP) - FL (MEHHP, MEOHP, ∑DEHP)

^1^Unless otherwise specified, studies included term, preterm, parous, and nulliparous births, and models of birth outcomes adjusted for or standardized to gestational age.

^2^Blank cells indicate associations were not examined; NS indicates that associations within that category were examined but not statistically significant at p<0.05.

*Average of two or more urine samples.

Abbreviations. AC, abdominal circumference; BPD, biparietal diameter; EFW, estimated fetal weight; FL, femur length; GA, gestational age; HC, head circumference; IUGR, intrauterine growth restriction, defined as estimated fetal weight below the 10^th^ centile for gestational age; MBP, monobutyl phthalate; MBzP, monobenzyl phthalate; MCOP, monocarboxy-isooctyl phthalate; MCNP, monocarboxy-isononyl phthalate; MCPP, mono-3-carboxypropyl phthalate; MEP, monoethyl phthalate; MECPP, mono-2-ethyl-5-carboxypentyl phthalate; MEHHP, mono-2-ethyl-5-hydroxyhexyl phthalate; MEHP, mono-2-ethylhexyl phthalate; MEOHP, mono-2-ethyl-5-oxohexylphthalate; MiBP, monoisobutyl phthalate; MMP, monomethyl phthalate; MnBP, mono-*n*-butyl phthalate; ∑DEHP, molar sum of the di-2-ethylhexyl phthalate metabolites that follow; ∑HMW, molar sum of the high molecular weight phthalate metabolites that follow; ∑LMW, molar sum of the low molecular weight phthalate metabolites that follow.

Two small hospital-based case-control studies from the same research group reported that levels of DEHP metabolites measured at a single time point in the third trimester were associated with increased odds of IUGR or “fetal growth restriction” (diagnosis of either IUGR or low birth weight) [97, 126]. A study restricted to male infants measured phthalate metabolite concentrations in first morning urine voids collected from women between 22 and 29 gestational weeks and measured fetal growth by ultrasound at 12.6, 22.5, and 32.6 weeks gestation [99]. Urinary concentrations of high molecular weight phthalate metabolites – including MBzP, monocarboxy-isononyl phthalate (MCNP), and metabolites of DEHP – were statistically significantly inversely associated with both biparietal diameter and estimated fetal weight throughout pregnancy. MCNP, however, was significantly positively associated with ultrasound measures of femur length during gestation. Another European study averaged phthalate concentrations in maternal urine samples collected at 12 and 32 weeks of gestation and measured fetal size and growth rates from ultrasounds collected at 12, 20, and 34 weeks of pregnancy [100]. While they found inverse associations between mono-*n*-butyl phthalate (MnBP, a metabolite of DBP) and fetal size and growth rates early in pregnancy (at and between 12 and 20 weeks gestation), they report positive associations between MBzP and MnBP and the rate of fetal growth between 20 and 34 weeks of gestation.

Finally, we previously examined ultrasound measures of fetal growth at up to three times per participant during pregnancy and phthalate measures collected at up to four times in pregnancy [101]. Although phthalate metabolite concentrations were not significantly associated with birth weight, cumulative exposure to high molecular weight phthalate metabolites (notably MBzP and metabolites of DEHP) over pregnancy was significantly negatively associated with head circumference, abdominal circumference, femur length, and estimated fetal weight. MEP was associated with reduced head circumference in female fetuses only.

#### Summary

While there is a large and growing base of literature exploring the relationship between gestational exposure to phthalates and fetal growth, the relationship remains in question. Studies that combined two or more samples of urinary phthalate metabolites collected during pregnancy found few associations with birth weight or other growth outcomes measured at birth [100, 101, 105, 106, 109–112]. In studies measuring fetal growth during gestation via ultrasound, metabolites of high molecular weight phthalates, particularly DEHP metabolites and MBzP, appeared to be related to perturbations in fetal growth. However, these studies were limited in number and inconsistent in both methodology and results reported.

### Environmental phenols and other non-persistent consumer product chemicals

Phenolic compounds are used in thousands of consumer and industrial products, and human exposure to some of these chemicals is essentially ubiquitous throughout high income countries [10, 127–129]. BPA is considered a high production volume chemical, and more than 1 million pounds of BPA are released into the environment annually [130]. It is used in the manufacturing of polycarbonate plastics and in epoxy resins and is often found in a variety of consumer products such as plastic bottles, children’s toys, inner coatings of food packaging, dental sealants, automobiles, and paper used in register receipts [131, 132]. Parabens are added to foods and widely used as preservatives in cosmetics, personal care products, and pharmaceuticals [133]. Benzophenone-3 (2-hydroxy-4-methoxybenzophenone, oxybenzophenone, oxybenzone [BP3]) absorbs and scatters ultraviolet radiation and is used in sunscreens and other personal care products as well as food packaging [134]. Triclosan (5-chloro-2,4-dichlorophenoxy) phenol [TCS]) and triclocarban (3,4,4-Trichlorocarbanilide [TCC]) are chemicals used as antimicrobial and antibacterial agents in personal care products and consumer products such as disinfectant, soaps, and cleaning products, deodorants, toothpastes, and plastic additives [135–137]. Chlorophenols such as 2,4-dichlorophenol (2,4-DCP) and 2,5,-dichlorophenol (2,5-DCP) and their precursors are used in the production of agricultural and pharmaceutical products including herbicides, antiseptics, antimicrobial agents, deodorizers [138]. Organophosphorous compounds are commonly used as flame retardants in furniture, baby products, electronics, and construction materials, and as an additive in rubbers, plastics, and some personal care products [139–142]. Given the variety of products in which these chemicals exist, pathways of exposure to humans vary. Exposure to BPA, for example, is largely through the diet, while exposure to BP3 is likely due primarily to dermal application of products containing these compounds.

#### Environmental phenols and other non-persistent consumer product chemicals and fetal growth outcomes measured at birth

We identified 27 studies conducted in the U.S., Europe, and Asia that examined associations between environmental phenols, parabens, or organophosphate ester flame retardants in maternal urine samples collected during pregnancy to at least one infant size outcome measured at birth (Table 3). As with studies of phthalates, the studies varied by sample size (from fewer than 200 infants to 1100), timing of exposure (urine collected preconception [98, 161], at time points throughout pregnancy [89, 96, 100, 101, 108, 110, 143, 145, 146, 149, 151, 152, 155, 156, 158, 160, 161], or as late as day of delivery [108, 144, 147, 148, 150, 153, 154, 159, 162, 163]), chemicals measured, outcomes measured, covariates included in models, statistical methodology used, and associations reported.

**Table 3:** Studies of maternal prenatal urinary biomarkers of phenols^1^ and fetal growth outcomes measured at birth

Reference	Country, years	N	Urinary phenol(s)	Timing of urine collection	Birth size outcome(s)	Notes^2^	All^3^	Girls^3^	Boys^3^
Berkowitz et al. 2004 [143]	USA 1998-2002	404	PCP	3^rd^ trimester	BW BL HC	primiparous only	NS	NS	NS
Wolff et al. 2008 [89]	USA 1998-2002	404	2,4-DCP, 2,5-DCP, BP3, BPA, TCS	3^rd^ trimester	BW BL HC	primiparous only	NS	NS	- BW (2,5-DCP) + BW (BP3) - BL (2,5-DCP)
Philippat et al. 2012 [96]	France 2003-2006	191	2,4-DCP, 2,5-DCP, BP3, BPA, TCS ∑PB: BuPB, EtPB, MePB, PrPB,	21 weeks	BW BL HC				- BW (2,4-DCP, 2,5-DCP) + BW (BPA) + HC (2,4-DCP, BPA, BP3)
Tang et al. 2013 [144]	China 2010-2012	567	BP3, BPA	Delivery	BW BL		NS		
Lee et al. 2014 [145]	Korea 2007-2010	757	BPA	3^rd^ trimester	BW BL HC		+ BW (BPA)	NS	+ BW (BPA) + BL (BPA)
Philippat et al. 2014 [146]	France 2003-2006	520	2,4-DCP, 2,5-DCP, BP3, BPA, BuPB; EtPB, MePB, PrPB, TCS	26 weeks	BW BL HC				NS
Huo et al. 2015 [147]	China 2012-2014	452	BPA	Delivery	LBW		- LBW (BPA)	- LBW (BPA)	NS
Smarr et al 2015 [98]	USA 2005-2009	233	BPA	Pre-conception	BW BL HC	not adjusted for GA	NS		
Casas et al. 2016 [100]	Spain 2004-2006	470	BPA	12, 32 weeks**	BW BL HC		NS	NS	NS
Ferguson et al. 2016 [101, 102]	USA 2006-2008	482	BPA	10, 18, 26, 35 weeks**	BW		NS	NS	NS
Guo et al. 2016 [148]	China 2009-2010	1100	2,4-DCP, 2,5-DCP, 2,4,5-TCP, 2,4,6-TCP, PCP	Delivery	BW BL HC		- BW (2,4,6-TCP, PCP) - HC (2,4,6-TCP, PCP)	- HC (2,5-DCP, 2,4-DCP)	- BW (2,4,6-TCP, PCP) - BL (PCP) - HC (2,4,6-TCP, PCP)
Lassen et al. 2016 [149]	Denmark 2010-2012	514	TCS	28 weeks	BW BL HC			NS	- HC (TCS)
Ding et al. 2017 [150]	China 2010-2013	496	BPA, TCS	Delivery	BW BL HC LBW SGA		NS	NS	+ BL (BPA)
Etzel et al. 2017 [151]	USA 2003-2006	378	TCS	16, 26.5 weeks**	BW BL HC LBW SGA	not adjusted for GA	- BW (TCS)	NS	NS
Geer et al. 2017 [152]	USA 2007-2009	185	2’-OH-TCC, 3’-OH-TCC, 3’-Cl-TCC, BePB, BuPB, EtPB, MePB, PrPB, TCS, TCC	3^rd^ trimester	BW BL HC LBW	not adjusted for GA	+ LBW (3’-Cl-TCC)		
Huang et al. 2017 [108]	Taiwan 2010	162	BPA	11 weeks	BW BL HC		NS		
				26 weeks			NS		
				Delivery			- HC (BPA)		
Wu et al. 2017 [153]	China 2012-2014	1006	∑PB: BePB, BuPB, EtPB, MePB, PrPB,	Delivery	BW BL		NS	NS	+ BL (MePB)
Wang et al. 2017 [154]	China 2012-2013	620	BPA	Delivery	BW		NS		
Woods et al. 2017 [110]	USA 2003-2006	272	BPA	16, 26 weeks**	BW		NS		
Lee et al. 2018 [155]	Korea 2006-2010	788	BPA	36 weeks	BW BL		+ BW (BPA)	NS	+ BW (BPA)
Ferguson et al. 2018 [156, 157]	USA 2006-2008	482	2,4-DCP, 2,5-DCP, BP3, BPS, BuPB, EtPB, MePB, PrPB, TCS, TCC	10, 18, 26, 35 weeks**	BW		NS	- BW (2,4-DCP, 2,5-DCP) - BL (2,4-DCP)	NS
Hoffman et al. 2018 [158]	USA 2001-2006	349	BDCIPP, DPHP, ip-PPP, BCIPH-IPP, BCIPP, tb-PPP	27 weeks	BW	not adjusted for GA		- BW (ip-PPP)	NS
Huo et al. 2018 [159]	China 2012-2014	1006	TCS	Delivery	BW BL LBW		NS	NS	NS
Krause et al. 2018 [160]	Denmark 2012-2014	157	BP1, BP3	18 weeks	BW BL HC			NS	NS
Messerlian et al. 2018 [161]	USA 2005-2016	346	BP3, TCS ∑PB: BuPB, EtPB, MePB, PrPB	Pre-conception	BW HC	not adjusted for GA	- HC (MePB, ∑PB)	NS	- BW (PrPB)
				6, 21, 35 weeks**			NS	NS	NS
Ouyang et al. 2018 [162]	China 2012-2013	620	TCS	Delivery	BW		NS	+ BW (TCS)	NS
Wan et al. 2018 [163]	China 2012-2014	985	BPS	Delivery	BW BL SGA		NS	NS	NS

^1^Includes environmental phenols and other non-persistent consumer product chemicals

^2^Unless otherwise specified, studies included term, preterm, parous, and nulliparous births, and models of birth outcomes adjusted for or standardized to gestational age.

^3^Blank cells indicate associations were not examined; NS indicates that associations within that category were examined but not statistically significant at p<0.05. Note that (-) associations for low birth weight (LBW) and small for gestational age (SGA) indicate *decreased risk* for these outcomes, while (+) associations indicated *increased risk* with increased exposure.

*Average of two or more urine samples.

Abbreviations. 2’-OH-TCC, 2’-hyhroxy-TCC; 3’-OH-TCC, 3’-hyhroxy-TCC; 3’-Cl-TCC, 3,3',4,4'-tetrachlorocarbanilide; 2,4-DCP, 2,4-dichlorophenol; 2,5-DCP, 2,5-dichlorophenol; 2,4,5-TCP, 2,4,5-trichlorophenol; 2,4,6-TCP, 2,4,6-trichlorophenol; BePB, benzyl paraben; BL, birth length; BP1, benzophenone-1; BP3, benzophenone-3; BPA, bisphenol-A; BPS, bisphenol-S; BuPB, butyl paraben; BW, birth weight; EtPB, ethyl paraben; GA, gestational age; HC, head circumference, LBW, low birth weight; MePB, methyl paraben; PCP, pentachlorophenol; PrPB, propyl paraben; SGA, small for gestational age; TCC, triclocarban; TCS, triclosan; ∑PB, summary measure of the parabens that follow.

The most frequently measured environmental phenol was BPA. Most of the 14 studies we identified found no statistically significant associations between prenatal maternal urinary BPA and birth size outcomes [89, 98, 100, 101, 108, 110, 144, 146, 154]. In five studies, BPA was significantly related to increased size at birth [96, 145, 147, 150, 155]. All of these studies reported results of models restricted to male infants; four found statistically significantly positive associations between BPA exposure and birth size in boys [96, 145, 150, 155]. Notably, all three studies with multiple measures of urinary BPA during pregnancy found no relationship between gestational BPA and size at birth [100, 101, 110]. We found two studies of bisphenol S, a primary BPA alternative, with null findings [156, 163].

12 studies examined prenatal exposure to triclosan (TCS) and size at birth, nine of which found no statistically significant associations, including two which collected urinary biomarkers at 3-4 points during gestation [89, 96, 146, 148, 150, 152, 156, 159, 161]. However, researchers in Cincinnati, OH, found that TCS measured twice in pregnancy was associated with decreases in birth weight, length, and head circumference [151], and a large study in Denmark found that TCS measured at 28 weeks of gestation was significantly associated with decreased head circumference in boys [149].

Four of five studies measuring dichlorophenols found statistically significant inverse associations with birth size [89, 96, 148, 156]. Two found that levels of 2,5,-DCP and 2,4-DCP measured during pregnancy were associated with reduced birth weight and length in models restricted to male infants [89, 96], while two reported inverse associations between 2,5,-DCP or 2,4-DCP and birth weight, length, or head circumference in models restricted to female infants [148, 157], and a study of 1100 Chinese infants reported inverse associations between 2,4,6-TCP and pentachlorophenol measured at delivery and birth size in un-stratified models [148]. However, a study of 520 male infants in France found no relationship between gestational exposure to 2,5-DCP or 2,4-DCP and birth size, despite similar distributions of dichlorophenol concentrations to the other four studies [146]. Interestingly, although three of these studies evaluated effect measure modification by including sex*exposure interaction terms [89, 148, 156], each reported different results of these analyses. One found no significant modification of any effect by infant sex [148], and two found significant modification of the association between 2,5,-DCP or 2,4-DCP and birth weight or length, but in opposite directions by sex [89, 156].

There was minimal evidence of any association between gestational exposure to parabens or benzophenones and size at birth. Of six studies of parabens, four found no statistically significant associations with birth size [96, 146, 152, 156], including two cohorts restricted to male infants [96, 146]. Three studies included sex*paraben terms to evaluate effect measure modification by infant sex, but none found statistically significant interactions [153, 156, 161]. In one U.S. study, preconception levels of parabens were associated with reduced head circumference in the overall cohort and with reduced birth weight in models restricted to girls [161]. A single study found statistically significant effect measure modification by sex of the association between BP3 and birth weight, with BP3 associated with increased birth weight in boys but not in girls [89]. A study restricted to male infants in France additionally reported a positive association between prenatal BP3 exposure and head circumference [96]; however five others found no relationship between BP3 and birth size [144, 146, 156, 160, 161].

Triclocarban (TCC) and non-persistent flame retardants have not been well studied in this field. Two U.S. studies found generally null associations between gestational TCC and birth size outcomes [152, 156]. A single recent study evaluated relationships between prenatal maternal urinary organophosphate flame retardant chemicals and birth weight but found minimal evidence of associations [158]. Of the six chemicals measured, a single phosphate – isopropyl-phenyl phenyl phosphate (ip-PPP) – was significantly associated with reduced birth weight in girls.

We additionally identified nine studies that measured environmental phenols and other non-persistent chemicals in media other than maternal prenatal urine, with mixed results (Additional file 2: Table S2). Five studies measured BPA at delivery in maternal or cord blood, plasma, and/or serum, or in the placenta, with generally null or positive associations with infant birth size [164–168]. Three studies measured BPA early in pregnancy in maternal blood [167, 169] and in amniotic fluid [170], two of which found inverse associations between BPA levels and birth size. One small U.S. study of environmental phenols in cord plasma found inverse associations between paraben levels and birth length, and a small study in Denmark reported inverse, non-monotonic associations between a benzophenone measured in maternal serum at 18 weeks gestation and birth size in boys [160]. These results should be interpreted with caution, however, as conjugated BPA measured in urine is considered the most valid biomarker of human exposure, while other matrices are more susceptible to contamination and mismeasurement [84].

#### Environmental phenols and other non-persistent consumer product chemicals and fetal growth outcomes measured during gestation

Six studies of five populations in the U.S., Europe, and Korea examined associations between maternal prenatal environmental phenol or other non-persistent consumer product chemical levels and *in utero* growth outcomes (Table 4) [100, 101, 146, 155, 156, 171]. All measured BPA; however, associations between prenatal BPA and fetal growth assessed by ultrasound were inconsistent. A repeated measures analysis of three urine samples and two ultrasound measures in the Netherlands reported inverse associations between BPA levels and both estimated fetal weight and head circumference [171]. A study of male infants in France found no association between BPA measured at 26 weeks and ultrasound measures of fetal growth at multiple time points in pregnancy [146]. Although a study in Spain found some statistically significant inverse associations between BPA measured twice in pregnancy and ultrasound measures of femur length and estimated fetal weight, these associations were not robust in sensitivity analyses [100]. Furthermore, we previously examined repeated measures of maternal urinary BPA during pregnancy and concluded there was no evidence to support an association with fetal growth [101]. Finally, a single recent cross-sectional study of third trimester BPA and fetal size reported an inverse association between BPA and femur length assessed by ultrasound [155].

**Table 4:** Studies of maternal prenatal urinary biomarkers of phenols^1^ and fetal growth outcomes measured *in utero*

Reference	Country, years	N	Urinary phenol(s)	Timing of urine collection	*in utero* outcome(s)	Timing of outcome assessment	All^2^	Girls^2^	Boys^2^
Snijder et al. 2013 [171]	the Netherlands 2004-2005	219	BPA	13.2, 20.7, 30.4 weeks+	EFW HC	20.5, 30.2 weeks	- EFW (BPA) - HC (BPA)		
Philippat et al. 2014 [146]	France 2003-2006	520	2,4-DCP, 2,5-DCP, BP3, BPA, BuPB, EtPB, MePB, PrPB, TCS	26 weeks	EFW AC BPD HC FL	12 weeks			NS
						22 weeks			NS
						32 weeks			- EFW (TCS) + HC (2,5-DCP) - AC (TCS)
						growth rate 22-32 weeks			- EFW (TCS) - AC (TCS)
						growth rate 32 weeks-birth			+ EFW (TCS, PrPB)
Casas et al. 2016 [100]	Spain 2004-2006	470	BPA	12, 32 weeks*	EFW AC BPD HC FL	12 weeks	NS	+ EFW (BPA) + AC (BPA)	NS
						20 weeks	NS	NS	- FL (BPA)
						34 weeks	NS	NS	NS
						growth rate 12-20 weeks	- FL (BPA)	NS	- EFW (BPA) - FL (BPA)
						growth rate 20-34 weeks	NS	NS	NS
Ferguson et al. 2016 [101, 102]	USA 2006-2008	482	BPA	10, 18, 26, 35 weeks*	EFW AC HC FL	26, 35, 38 weeks	NS	NS	NS
Ferguson et al. 2018 [156, 157]	USA 2006-2008	482	2,4-DCP, 2,5-DCP, BP3, BPS, BuPB, EtPB, MePB, PrPB, TCC, TCS	10, 18, 26, 35 weeks*	EFW AC HC FL	26, 35, 38 weeks	NS	- EFW (2,5-DCP) - AC (BP3, MePB, EtPB, PrPB) + FL (BPS)	- EFW (BPS)
Lee et al. 2018 [155]	Korea 2006-2010	788	BPA	36 weeks	EFW FL	3^rd^ trimester	- FL (BPA)	NS	NS

^1^Includes environmental phenols and other non-persistent consumer product chemicals. All studies included term, preterm, parous, and nulliparous births, and models of birth outcomes adjusted for or standardized to gestational age.

^2^Blank cells indicate associations were not examined; NS indicates that associations within that category were examined but not statistically significant at p<0.05.

+Results only statistically significant among women with three urinary BPA measurements during pregnant (*n*=80)

*Average of two or more urine samples.

Abbreviations. 2,4-DCP, 2,4-dichlorophenol; 2,5-DCP, 2,5-dichlorophenol; AC, abdominal circumference; BP3, benzophenone-3; BPA, bisphenol-A; BPD, biparietal diameter; BPS, bisphenol-S; BuPB, butyl paraben; EFW, estimated fetal weight; EtPB, ethyl paraben; FL, femur length; HC, head circumference; MePB, methyl paraben; PrPB, propyl paraben; TCC, triclocarban; TCS, triclosan

We previously observed statistically significant effect measure modification and sex-specific associations between repeated measures of other environmental phenols and non-persistent consumer product chemicals and fetal growth measured *in utero* [156, 157]. Detection of BPS in prenatal urine was inversely associated with estimated fetal weight in boys and positively associated with femur length in girls [157]. 2,5-DCP, BP3, and parabens were inversely associated with repeated measures of fetal size in girls [157]. We found no associations with TCS, in contrast to the French study of male infants which found that TCS was associated with reduced fetal size at 32 weeks gestation [146].

#### Summary

BPA is the most commonly studied chemical in research examining prenatal exposure to environmental phenols or other non-persistent consumer product chemicals and fetal growth or birth size outcomes. Studies of birth size and of fetal size measured by ultrasound provide limited support for an association between prenatal exposure to this phenol and fetal growth. Similarly, a recent meta-analysis concluded that maternal prenatal BPA exposure was positively, though not statistically significantly, associated with birth weight [172]. However, growing evidence suggests that exposure to dichlorophenols during pregnancy may be related to reduced fetal growth. Although exposure to dichlorophenols and parabens is prevalent in North American, European, and Asian populations, there is limited research regarding gestational exposure to these chemicals and effects on fetal development. Studies that utilize multiple measures of exposure during pregnancy and evaluate fetal growth during gestation would further develop and potentially strengthen this evolving literature.

### Non-persistent pesticides

Almost 6 billion pounds of pesticides were used globally in 2012, with 20% of that application occurring in the U.S. [173]. Herbicides – such as glyphosate (RoundUp**®**), atrazine, Metolachlor-S, and 2,4-Dichlorophenoxyacetic acid (2,4-D) – account for approximately half of the world’s pesticide usage [173]. An estimated 78 million U.S. households used pesticides in 2007 [174]. In the 1990s, organophosphate pesticides (OPPs) accounted for approximately two thirds of insecticides used in the U.S. [174]. In humans, 75% of OPPs metabolize and are excreted in urine as dialkyl phosphates (DAPs). Urinary DAP metabolites may not be an accurate measure of direct pesticide exposure, as they can reflect exposure to OPPs or to the non-toxic DAPs themselves, as OPPs can degrade into DAP metabolites in the environment as well [175, 176]. As indoor residential use of OPPs has decreased dramatically in the U.S. over the past two decades, carbamate and pyrethroid insecticides have replaced them in home and garden applications [173, 174, 177].

#### Non-persistent pesticides and fetal growth outcomes measured at birth

We identified 17 studies that evaluated associations between gestational exposure to biomarkers of non-persistent pesticides and birth size outcomes, the majority of which measured metabolites of organophosphate pesticides (Table 5). Nearly all of these twelve studies of eight populations in the U.S., China, Thailand, Taiwan, and Denmark measured DAPs, non-specific metabolites of OPPs, with mostly null results. While one New York City study reported inverse associations between prenatal DAPs and head circumference measured at birth [178], another study in central California found the opposite [180]. One U.S. study and one study in Taiwan reported significant inverse associations between prenatal DAPs and birth weight [108, 182]. Researchers in China also found inverse associations between DAPs and head circumference, particularly among boys but not in girls [188].

Several studies found statistically significant modification of associations between prenatal DAPs and birth size by *PON1* genotype and status [178, 181, 182, 186, 187]. Single nucleotide polymorphisms at position 192(Q/R) and promoter region -108(C/T) in the *PON1* gene control the levels and efficiency of paraoxonase, an enzyme that can detoxify OP pesticides such as chlorpyrifos, parathion, and diazinon [192]. There appear to be three human *PON1* phenotypes – low, moderate, and high enzymatic activity – with low activity related to reduced detoxification. *PON1*_*192QQ*_ and *PON1*_*-108TT*_ genotypes generally correspond to low activity (and possibly greater vulnerability to adverse impacts of OPPs), while genotypes *PON1*_*192RR*_ and *PON1*_*-108CC*_ correspond to high activity [187, 192]. However, modification of OPP associations with fetal growth by these genotypes has not been consistent across studies. Two studies found inverse associations between DAP levels and birth outcomes in infants whose mothers had with low enzymatic activity or the *PON1*_*192QQ*_ genotype [178, 186]; another found inverse associations between birth weight and DAP levels in infants with the heterozygous *PON1*_*192QR*_ and *PON1*_*-108CT*_ genotypes [182]; while a study of California farmworkers found positive associations between prenatal DAP levels and infant size at birth among infants with high enzymatic activity or the *PON1*_*192RR*_ or *PON1*_*-108CT*_ genotypes [181]. Most of these findings were not reproduced in a pooled study (see Table 5 note), which instead found inverse associations between prenatal dimethylphosphate levels and birth length in infants with the *PON1*_*192RR*_ genotype and positive associations between prenatal dimethylphosphate levels and birth length in infants with the *PON1*_*-108TT*_ genotype [187]. This large pooled study also found statistically significant modification by maternal race, where prenatal DAP levels were inversely associated with infant size at birth in non-Hispanic black women [187].

Four studies of three different cohorts measured urinary metabolites specific to individual OPPs [143, 178, 180, 189]. There were mostly null findings in any of the overall populations, except for a positive association between prenatal 4-nitrophenol (a metabolite of methyl parathion, parathion, and other non-pesticide chemicals) and birth length among children of farmworkers in central California [180]. Additionally, a New York City study found a significant inverse association between prenatal TCPy levels (3,5,6-trichloro-2-pyridinol, a metabolite of chlorpyrifos and chlorpyrifos methyl**)** and head circumference in infants whose mothers had low PON1 enzymatic activity [143].

Four studies measured urinary metabolites of pyrethroid pesticides, the most commonly-measured of which was 3-phenoxybenzoic acid (3-PBA), with mixed results [143, 184, 185, 189]. While a Japanese study found positive associations between early levels of 3-PBA early in gestation and infant size at birth [184], a Danish study found mostly null associations between 3-PBA measured in mid pregnancy and birth outcomes [189]. A study of Chinese infants found an inverse association between total urinary pyrethroid metabolites measured at delivery and birth weight [185].

Other non-persistent pesticides measured in prenatal urine included the herbicide 2,4-D [189], the carbamate pesticide carbofuran [191], the herbicide atrazine [179], glyphosate, a broad spectrum herbicide [190], and the chloroacetanilide herbicides alachlor, metolachlor, and acetolachlor [179]. A large cohort study in France found that prenatal atrazine metabolite levels were associated with increased risk of being in the lowest 5^th^ percentile for birth weight and for head circumference at birth, while prenatal metolachlor levels were inversely associated with head circumference measured at birth [179].

Five studies also measured biomarkers of prenatal exposure to non-persistent pesticides in other matrices (Additional file 2: Table S3). Four of these studies utilized umbilical cord blood [193–197]. One also measured pesticides in maternal serum at delivery [193], and a study in Greece measured DAPs in amniotic fluid collected at 16-20 weeks gestation [198]. Notably, we were unable to find any studies of prenatal exposure to non-persistent pesticides and fetal growth measured during gestation.

#### Summary

Despite evidence of widespread use of and exposure to non-persistent pesticides in the U.S., Europe, and Asia, there is little research regarding the effects of prenatal exposure to these chemicals on fetal growth. Use of the most well-studied chemicals, OPPs, has decreased substantially in the U.S. over the past two decades, but there appears to be little epidemiologic information regarding how replacements like pyrethroid pesticides may affect fetal growth measured at birth, and no studies to date examining associations with fetal growth measured during gestation via ultrasound.

## Limitations and Research Gaps

As described previously, based on known biological pathways to fetal growth and evidence from animal models, it is highly plausible that gestational exposure to non-persistent chemicals perturbs fetal growth in humans. However, the epidemiologic evidence for such associations is inconsistent. Because relatively few studies exist, and because of the particular complexity in evaluating the relationships between non-persistent chemicals and fetal growth, we believe that many current studies are insufficiently powered or inadequately designed to detect effects. In our review of the environmental epidemiology literature, we encountered several areas of inconsistency in methods used to evaluate associations between gestational exposure to non-persistent chemicals and fetal growth. Below, we summarize key methodological limitations that likely contribute to conflicting conclusions and make recommendations to aid investigators planning further research.

### Exposure biomarkers

#### Variability over time and the need to assess windows of vulnerability

Pregnancy is a period of rapid physiological and behavioral change. Both exposure opportunities and vulnerability to physiological/biological effects of exposure to non-persistent chemicals may vary from preconception to delivery. Moreover, the half-lives in the body of these chemicals is a matter of hours to weeks. Exposure to sources of phthalates, environmental phenols, parabens, organophosphate ester flame retardants, and non-persistent pesticides can vary throughout a single day or week, with some compounds being excreted within hours of exposure [199, 200]. A single biomarker, while perhaps an accurate representation of exposure over the past few hours, may not reflect exposure to a non-persistent chemical over the course of pregnancy. This is exemplified by an extensive literature on intraclass correlation coefficients (ICCs) for non-persistent compounds measured in single spot urine samples during pregnancy. ICCs tend to be higher for metabolites that come from personal care products or materials found in the home (e.g. MEP, MBzP) than for metabolites for which the likely source of exposure is dietary (e.g. BPA, DEHP) [200–203]. Thus, relying on a single spot urine measurement of a non-persistent chemical can induce bias in its estimated effect, with as much as 40% attenuation in the effect estimate even with an ICC as high as 0.60 [204]. Measuring concentrations of a chemical in a 24 hour urine sample is more representative of the day’s exposure compared to a spot urine sample [205, 206]. First morning void samples are more complicated because time of day is a significant predictor of levels of phthalates and BPA in urine, with higher levels of BPA and high molecular weight phthalates observed in samples collected in the evening, and highest levels of MEP in the morning [201, 202, 207].

Because of this variability, measurement of exposure biomarkers in multiple specimens collected across pregnancy is recommended. The majority of research we identified collected a biomarker of exposure at a single time point in pregnancy, ranging in gestational time from preconception to delivery. As exemplified by Snijder et al. [171], where statistically significant effect estimates were only observed among women with three or more BPA measurements, utilizing more than one urine specimen for exposure assessment can improve ability to detect effects. Measuring more than one sample of urine collected at different times of day, particularly relative to timing of a participant’s most recent meal or urination, can improve exposure characterization of chemicals with dietary sources [200, 201].

Researchers may be reluctant to measure numerous biomarkers during pregnancy due to high cost of laboratory assays. Within-subject pooling of biospecimens, where samples from a single individual at multiple time points are combined prior to measurement, can be used to address this concern while also reducing misclassification of exposure assessment [204]. Increasing the number of biospecimens in an individual’s pooled assay can both decrease bias in the effect estimate and increase power [204]. Additionally, within-subject pooling can improve exposure characterization over first morning voids [202]. At least 6 and 35 specimens are required to limit bias to 10% attenuation for chemical with ICC of 0.6 and 0.2, respectively, though [204]. This number of biospecimens may be unfeasible to collect for logistical or financial reasons. However, if the same number of biospecimens are pooled for each participant, and reliable estimates of ICCs are available, *a posteriori* disattenuation correction can virtually eliminate bias in effect estimates [204]. Moreover, if at least two biospecimens are measured separately, measurement error models such as simulation extrapolation or regression calibration can be used to reduce bias to less than 10% [204].

An important limitation to pooling samples across weeks of pregnancy, however, is that key windows of vulnerability to exposure may be missed. Consider a chemical for which exposure during the first trimester is the most relevant for fetal growth and for which there is high variability (low ICC) across pregnancy. If the urine sample from this time point is pooled with those collected later in pregnancy, any potential associations would be diluted. Moreover, in studies with biomarkers and growth measures collected at multiple time points, it is sometimes unclear whether exposures truly precede outcomes. When samples are pooled or exposure levels are averaged across multiple samples it is possible that some of the individual samples were actually collected after the time point when growth was assessed, which violates the temporality assumption, i.e., that the measured exposure precedes the outcome of interest. It is important that researchers therefore consider and clearly convey when biological samples were collected in relation to when growth outcomes were measured.

Thus, in an ideal setting, repeated urine samples would be collected within trimesters and across gestation and analyzed individually. Since this is not always feasible financially, alternative approaches—such as exploring windows of vulnerability in a subset and then subsequently pooling—are encouraged. Investigators should carefully consider the time period of exposure that one or more biomarkers reflect, as well as hypotheses regarding mechanisms of action when designing exposure assessment methods for large studies. It should be noted that the windows of exposure measured were highly variable across the literature reviewed here. If the growth of the fetus is more vulnerable to environmental stressors during one point in gestation than another, this variation likely contributes to the lack of consistency seen in results. While we did not formally evaluate whether associations were more consistent when biomarkers were measured earlier versus later in pregnancy, we observed no clear patterns in associations by timing of exposure assessment.

In summary, careful consideration in study design must be given to determining the mode of urine sample collection, number of specimens, and whether or not to pool. Striking a balance between cost, participant burden, and scientific integrity can be challenging in this field.

#### Other issues with exposure biomarkers

Many studies included in this review measured exposure to phthalates, environmental phenols, or other non-persistent consumer products at delivery [106, 108, 112, 113, 144, 147, 148, 150, 153, 154, 159, 162, 163, 183, 186, 188], and it was not always clear at what point during delivery urine samples were collected. This timing of exposure should be interpreted with caution. Phthalates are often present in medical devices, intravenous tubing, and medication coating, for example [85]. Exposure to these products prior to urine collection could produce higher urinary concentrations of these chemicals or these metabolites, but could not have a causal effect on fetal size at birth. Moreover, the single study that measured phthalate metabolites at delivery (specifically, prior to IV insertion) as well as earlier in gestation reported poor correlation between the two measures for all phthalates, but particularly for DEHP metabolites [106]. Even assuming these measures are uncontaminated, they still may not be representative of earlier, perhaps more relevant, windows of exposure in pregnancy.

Another limitation is the use of inappropriate biological matrices for measuring exposure. Urine is the preferred matrix for assessment of exposure to non-persistent chemicals, particularly at low concentrations [13]. Levels of parabens, environmental phenols, and metabolites of BPA and phthalates are orders of magnitude lower in blood than in urine, and true variation in exposure levels can be undetectable or masked by even very small amounts of contamination [84, 208]. While our review focused on studies which measured biomarkers of non-persistent chemicals in urine, we also identified over two dozen papers which utilized an alternative biomarker (see Additional file 2 “Supplemental tables”). Such studies are still informative and can add to the weight of evidence in favor of associations between non-persistent environmental toxicants and fetal growth. However, they are difficult to compare directly to studies of urinary biomarkers and should be interpreted with caution.

Finally, variation in levels of exposure to non-persistent environmental chemicals, as well as differences in the susceptibility of populations under study, can contribute to differences in the true effect between studies of the same exposure and outcome. Reporting the concentrations of every chemical measured in every study described is beyond the scope of this review. Rather, we recommend that researchers compare chemical exposure levels in their study population to those in both other study populations and in population-based samples (such as the National Health and Nutrition Examination Survey in the U.S.) to facilitate evaluation of these possible differences. Moreover, there is evidence that exposure to some non-persistent chemicals – such as BP3, TCS, and organophosphate pesticides – follow seasonal patterns [209–212]. Birth weight also follows seasonal patterns, though these patterns can vary by population and years under study [213–215]. Researchers should critically evaluate (using, for example, directed acyclic graphs [216]), whether season of measurement should be considered in modeling effects of non-persistent chemicals on fetal growth outcomes.

### Outcome assessment

The majority of the research we identified examined infant size measured at birth as a reflection of fetal growth in utero. While birth weight, for example, is a reliable metric, it is an incomplete measure of whether a fetus attained (or surpassed) its potential growth over the course of gestation. Other indices of suboptimal fetal growth, as established by the American College of Obstetricians and Gynecologists, include small for gestational age (SGA; <10^th^ percentile birth weight for gestational age at delivery) and intrauterine growth restriction, also referred to as fetal growth restriction (IUGR; <10^th^ percentile estimated fetal weight for gestational age at ultrasound scan) [217]. These measures are subject to measurement error from gestational age estimation and, for IUGR, from the measurement error in the ultrasound estimates of fetal weight. Additional error arises from individual differences in optimal weight. In other words, based on maternal, paternal, and environmental characteristics, ideal fetal or infant weight varies substantially across the population. This may be partially addressed by creating standardized curves based on one or more of these characteristics (e.g., the recent racial/ethnic group-specific curves developed by Buck Louis et al. [218] or customized growth curves [219]), but including all of the factors that influence diversity in ideal fetal size is not feasible.

Fetal size measured via ultrasound at multiple time points in pregnancy can reveal deviations from optimal fetal growth trajectories that would not be captured in a study of birth weight alone. Moreover, multiple ultrasound measures of fetal size during pregnancy can help inform how and when an exposure may have altered fetal growth. As reviewed above, there are a number of potential mechanisms by which non-persistent chemicals may perturb normal fetal growth during gestation. However, relatively few studies have collected both urinary biomarkers of non-persistent chemicals during gestation and *in utero* fetal growth outcomes (see Tables 2 and 4). Among these, there was minimal similarity in when and how often fetal size was determined, ranging from a single ultrasound measure at 36 weeks gestation [155] to three ultrasounds performed at 12-38 weeks gestation [99–101, 146, 156]. Three studies additionally examined associations between biomarkers of non-persistent chemicals and growth between ultrasounds (e.g., fetal growth rate between 12 and 20 weeks gestation) [99, 100, 146]. The lack of similarity in timing of ultrasound measurements makes comparing study results challenging. It may be particularly important in research studies of fetal growth to capture at least two measurements from the second half of pregnancy, when the most growth occurs. Our previous work has demonstrated that ultrasound measures taken later in pregnancy may be the most relevant for capturing associations with phthalate and phenol exposure [101, 156].

**Table 5:** Studies of maternal prenatal urinary biomarkers of pesticides and fetal growth outcomes measured at birth

Reference	Country, years	N	Urinary pesticides	Timing of urine collection	Birth size outcome(s)	Notes^1^	Results^2^	Interaction with maternal or child PON1 genotype or phenotype
Berkowitz et al. 2004 [143]	+USA, 1998-2002	404	TCPy, 3-PBA	3^rd^ trimester	BW BL HC	primiparous only		Low maternal AREase activity - HC (TCPy)
Wolff et al. 2007 [178]	+USA, 1998-2002	404	MDA ∑DMP: DMP, DMDTP, DMTP ∑DEP: DEP, DEDTP, DETP ∑DAP: DMP, DMDTP, DMTP, DEP, DEDTP, DETP	3^rd^ trimester	BW BL HC	primiparous only	- HC (∑DAP)	Low maternal AREase activity - BL (∑DMP) Maternal PON1_192_ QQ: - BW (∑DEP)
Chevrier et al. 2011 [179]	France, 2002-2006	579	atrazine, atrazine mercapturate, simazine, simazine mercapturate, desethylatrazine, desisopropyl atrazine, 2-chlorodiaminoatrazine, hydroxyatrazine, hydroxysimazine, hydroxydesethylatrazine, hydroxy-desisopropyl atrazine, hydroxy-2-chlorodiaminoatrazine (ammeline), alachlor, metolachlor, acetolachlor, 2,6-diethylaniline	1^st^ trimester	BW BL HC		- HC (metolachlor)	
Eskenazi et al. 2004 [180]	+USA, 1999-2000	488	MDA, TCPy, PNP, DEAMPY, IMPY, CMHC, CIT ∑DMP: DMP, DMDTP, DMTP ∑DEP: DEP, DEDTP, DETP ∑DAP: DMP, DMDTP, DMTP, DEP, DEDTP, DETP	13, 26 weeks**	BW BL HC		+ BL (PNP) + HC (∑DAP)	
Harley et al. 2011 [181]	+USA, 1999-2000	467	∑DMP: DMP, DMDTP, DMTP ∑DEP: DEP, DEDTP, DETP ∑DAP: DMP, DMDTP, DMTP, DEP, DEDTP, DETP	13, 26 weeks**	BW BL HC			Child PON1_-108_ CT: + BW (∑DMP, ∑DAP) Child PON1_192_ RR: + BW (∑DEP) High child AREase activity: + HC (∑DMP, ∑DAP)
Rauch et al. 2012 [182]	+USA, 2003-2006	306	∑DMP: DMP, DMDTP, DMTP ∑DEP: DEP, DEDTP, DETP ∑DAP: DMP, DMDTP, DMTP, DEP, DEDTP, DETP	16, 26 weeks**	BW	not adjusted for GA	- BW (∑DMP, ∑DAP)	Child PON1_-108_ CT: - BW (∑DAP) Child PON1_192_ QR: - BW (∑DAP)
Wang et al. 2012 [183]	China 2006-2007	187	DMP, DMTP, DEP, DETP, DEDTP	delivery	BW BL		NS	
Zhang et al. 2014 [184]	Japan 2009-2011	147	3-PBA	1^st^ trimester	BW BL HC		+ BW (3-PBA) + HC (3-PBA)	
Ding et al. 2015 [185]	China 2010	454	∑pyrethroids: cis-DCCA, trans-DCCA, 3-PBA	delivery	BW BL HC		- BW (∑pyrethroids)	
Naksen et al. 2015 [186]	Thailand 2011-2012	52	∑DMP: DMP, DMDTP, DMTP ∑DEP: DEP, DEDTP, DETP ∑DAP: DMP, DMDTP, DMTP, DEP, DEDTP, DETP	12 weeks	BW BL HC		NS	Low maternal PON1 activity: - BW (∑DAP) - HC (∑DMP, ∑DEP, ∑DAP) High maternal PON1 activity: + BW (∑DMP)
				32 weeks			NS	Low maternal PON1 activity: - HC (∑DEP, ∑DAP)
				delivery			NS	NS
Harley et al. 2016 [187]	USA (3 pooled studies) 1999-2006	1169	∑DMP: DMP, DMDTP, DMTP ∑DEP: DEP, DEDTP, DETP ∑DAP: DMP, DMDTP, DMTP, DEP, DEDTP, DETP	1-2 samples during pregnancy	BW BL HC		NS	Child PON1_-108_ TT: + BL (∑DMP) Child PON1_192_ RR: - BL (∑DMP) Maternal PON1_192_ QR: + HC (∑DEP)
Liu et al. 2016 [188]	China 2011-2012	310	∑DMP: DMP, DMDTP, DMTP ∑DEP: DEP, DEDTP, DETP ∑DAP: DMP, DMDTP, DMTP, DEP, DEDTP, DETP	delivery	BW BL HC		- HC (∑DAP)	
Woods et al. 2017 [110]	USA 2003-2006	272	∑DMP: DMP, DMDTP, DMTP ∑DEP: DEP, DEDTP, DETP ∑DAP: DMP, DMDTP, DMTP, DEP, DEDTP, DETP	16, 26 weeks**	BW		NS	
Huang et al. 2017 [108]	Taiwan 2010	105	∑DMP: DMP, DMDTP, DMTP ∑DEP: DEP, DEDTP, DETP ∑DAP: DMP, DMDTP, DMTP, DEP, DEDTP, DETP	11 weeks	BW BL HC		NS	
				26 weeks			- BW (DEP) - BL (DEP, ∑DEP)	
				delivery			NS	
Dalsager et al. 2018 [189]	Denmark 2010-2012	858	TCPy, 3-PBA, 2,4-D ∑DMP: DMP, DMDTP, DMTP ∑DEP: DEP, DEDTP, DETP ∑DAP: DMP, DMDTP, DMTP, DEP, DEDTP, DETP	28 weeks	BW HC		NS	
Parvez et al. 2018 [190]	USA 2015-2016	71	Glyphosate	11-39 weeks	BW HC		NS	
Zhang et al. 2018 [191]	China 2009-2010	1100	Carbofuran	delivery	BW BL HC		NS	

^1^Unless otherwise specified, studies included term, preterm, parous, and nulliparous births, and models of birth outcomes adjusted for or standardized to gestational age.

^2^Blank cells indicate associations were not examined; NS indicates that associations within that category were examined but not statistically significant at p<0.05.

*Average of two or more urine samples

+Included in Harley et al. 2016 pooled study

Abbreviations. 2,4-D, 2,4-dichlorophenoxyacetic acid; 3-PBA, 3-phenoxybenzoic acid; AREase, arylesterase; BL, birth length; BW, birth weight; cis-DCCA, cis-3-(2,2-Dichlorovinyl)-2,2-dimethylcyclopropane carboxylic acid; CIT, 5-chloro-1-isopropyl-3-hydroxytriazole; CMHC, 3-chloro-4-methyl-7-hydroxycoumarin; DEAMPY, 2-diethylamino-4-hydroxy-6-methylpyrimidine; DEP, diethylphosphate; DEDTP, diethyldithiophosphate; DETP, diethylthiophosphate; DMP, dimethylphosphate; DMDTP, dimethyldithiophosphate; DMTP, dimethylthiophosphate; HC, head circumference; IMPY, 2-isopropyl-4-methyl-6-hydroxypyrimidine; MDA, malathion dicarboxylic acid; PNP, 4-nitrophenol; TCPy, 3,5,6-trichloro-2-pyridinol; trans-DCCA, trans-3-(2,2-Dichlorovinyl)-2,2-dimethylcyclopropane carboxylic acid; ∑DAP, molar sum of the dialkyl phosphate metabolites that follow; ∑DEP, molar sum of the diethyl phosphate metabolites that follow; ∑DMP, molar sum of the dimethyl phosphate metabolites that follow

Another consideration in the analysis of ultrasound data is the approach for calculating standardized measurements (i.e., z-scores or centiles) for each measurement. Most studies apply population-specific references (e.g., the Generation R cohort, the LIFECODES birth cohort, and the INMA cohort) [100, 220–222]. However, alternative approaches, such as using customized growth curves (e.g., Buck-Louis et al. described above) or universal growth curves (e.g., INTERGROWTH-21^st^) are also options. While it is not clear what impact this choice has on associations between environmental exposures and fetal growth, this is a question worth investigating [223, 224].

We identified a single study that examined overgrowth as a potential adverse endpoint [166]. Macrosomia and large fetal size are related to a variety of adverse perinatal and longer-term health outcomes [225–227] and may reflect deviation from ideal fetal growth. Classifying overgrowth (for example, large-for-gestational-age births) as normal growth would fail to identify impacts of environmental toxicants that result in larger fetuses. To improve the understanding of when environmental exposures may influence fetal growth, further research should incorporate measures of fetal size during gestation to evaluate deviations – both decreases and increases – in growth trajectories over the course of pregnancy.

### Statistical approaches and bias

The epidemiologic literature regarding the potential effects of non-persistent environmental chemicals on fetal growth has increased substantially over the past decade. However, there remains variability in the statistical approaches employed by researchers in this field that is likely contributing to inconsistency and possibly bias in published effect estimates.

The majority of the research we identified used linear regression models of associations between continuous biomarker measures and continuous fetal size measures. These models assumed a monotonic, if not linear, relationship between exposures and outcomes. However, a number of studies that examined categories (tertiles or quartiles) of prenatal phthalate levels found few monotonic trends but identified non-monotonic statistically significant associations [98, 146, 160]. If physiological responses to these chemicals exist on a non-linear dose-response curve, it is possible that continuous linear regression models may be unable to detect real effects. We therefore recommend investigators examine non-linear and non-monotonic dose response curves. While categorical exposure variables are both simple to create and easy to interpret, they can be subject to limitations [228, 229]. Flexible approaches to assessing dose-response relationships, such as nonparametric regression, fractional polynomial regression, or the use of splines, may further improve assessment of the shape of dose response curves [228].

An additional consideration with respect to model selection is how to include repeated (non-independent) measures for an individual. We noted several methods employed by studies included in this review, including averaging measures from two or more time points to create a single exposure metric for the entire pregnancy [100, 105, 106, 109, 110, 151, 180–182], using linear mixed models to conduct repeated measures analyses [156, 171], evaluating measures collected at different time points in independent statistical models [106, 108, 109, 186], or examining cumulative averages [101]. These methods and others each have a number of benefits and limitations, and the most appropriate approach depends in part on whether the investigator aims to estimate average exposure over pregnancy or evaluate windows of vulnerability [230, 231].

Inclusion of covariates in statistical models differed greatly across the literature we identified. In particular, we noted variation in how studies incorporated gestational age in analyses of fetal size and evaluated potential modification or interaction by fetal sex. The majority of studies we identified adjusted models of fetal growth outcomes for gestational age. Researchers standardized *in utero* fetal size measures to gestational age at the time of ultrasound [101, 156] and to fetal growth curves created based on population or individual characteristics [100]. Some used linear mixed models that included random slopes for gestational age [99, 101, 156]. Many studies of infant size at birth (birth weight, birth length, etc.) reported results of regression models adjusted for gestational age at delivery. A number restricted results to term births only [104, 120–123, 126, 164, 169, 170, 185, 193, 197]. Several, however, reported results unadjusted for gestational age [98, 100, 104, 105, 107, 109, 112, 114, 118, 124, 151, 152, 154, 167, 168, 182]. There is clearly disagreement regarding the appropriateness of incorporating gestational age in models where the outcome of interest is fetal or birth size. Fetal size is largely a function of duration of gestation. However, gestational age at delivery may also be an intermediate variable on a casual path between an environmental exposure and birth weight; there is evidence that prenatal exposure to some non-persistent environmental chemicals may be related to reduced gestational age [144, 151, 182, 232–235]. Adjustment for gestational age in a model where the outcome is birthweight could therefore produce biased effect estimates [236, 237].

Maternal diet during pregnancy influences fetal growth and is also a primary source of exposure to some non-persistent chemicals [238]. Increased caloric intake during pregnancy is associated with increased birth weight [239], although there is evidence that consuming a diet high in processed or red meat, or high fat dairy, during pregnancy is associated with increased odds of giving birth to an SGA infant [240]. Eating canned food, fish, and fast food have also been shown to be positively correlated with BPA levels in pregnant women [241–243], and other bisphenols, such as BPS, have been detected in food as well [244]. Poultry, high-fat dairy, and fast food consumption may all be sources of exposure to phthalates such as DEHP [91, 245]. Dietary factors are often not well-characterized in environmental epidemiology studies and likely confound the relationship between prenatal exposure to some non-persistent chemicals and fetal growth. The limited or nonexistent control for these factors in statistical models or study design could explain some of the variability in the results among studies of chemicals for which diet is a primary source of exposure. Careful evaluation of these entangled relationships is therefore warranted.

There is inconsistency in the literature in whether researchers evaluate fetal sex as an effect measure modifying variable. Effect modification by fetal sex in this context deserves special consideration because of differences in: 1) how male and female fetuses grow and respond to the environment [246, 247]; 2) placental features that influence how chemicals are transferred and the dose of exposure to the fetus [248]; and 3) hormonal pathways and inflammatory responses that may be involved in mediating effects [39, 249]. While a number of studies reported results stratified by fetal sex (see Tables), others tested statistically for differences using interaction terms in regression models. These methods are not equivalent, and may result in different conclusions even in the same data [250]. We recommend an alternative augmented product term approach described by Buckley et al., which entails including both an exposure by sex product term and product terms for covariates by sex [250]. This method produces the same effect estimates as stratification but allows for formal statistical evaluation of heterogeneity using a Wald test or likelihood ratio test of the exposure by sex product term [250]. In this area of research, examination of sex differences should be standard, and methods for investigating those differences clearly relayed.

Reproductive-aged women are exposed to an unavoidable milieu of environmental chemicals that can be transferred to a developing fetus during pregnancy [251, 252]. While there is clear value in understanding if, when, and how maternal prenatal exposure to a single chemical may perturb normal fetal development, there is increasing interest in understanding how multiple chemicals or mixtures of exposures affect human health [253–255]. Although laboratory and statistical methods have made great strides in this field, their application in studies of non-persistent exposures and fetal growth has been limited. We identified only six studies that reported results of multipollutant models, each of which employed different statistical techniques to evaluate which chemical(s) in a mixture of exposures was/were most influential on birth size outcomes [100, 108, 110, 111, 116, 121]. In particular, Chiu et al. evaluated a variety of statistical approaches to assess effects of phthalate mixtures on birth weight [111]. Although none of the models produced statistically significant results, the authors highlighted the limitations of linear regression models in the presence of collinear exposures and high-dimensional correlation structures [111].

In addition to maternal exposures, paternal environmental exposures may also be related to fetal growth and development. While the focus of this review was maternal exposure to non-persistent chemicals, three studies included in this review additionally examined paternal exposure to non-persistent environmental exposures in the context of fetal growth [98, 109, 161]. As we begin to elucidate the mechanisms by which paternal chemical exposures affect fetal development, future epidemiologic research that examines multiple chemicals or classes of chemicals may provide a better understanding of how different profiles of environmental exposures interact to affect fetal health.

The studies we identified in this review included only live born infants in their analyses. If an environmental toxicant both acts to reduce fetal growth and increases risk of fetal demise, conditioning on live birth can lead to biased effect estimates [256]. The selection bias induced by excluding stillbirths and miscarriages can thus be conceptualized as conditioning on a collider in a directed acyclic graph [257]. This issue highlights the need for further investigation into the effects of non-persistent chemicals on early stages of pregnancy.

### Summary

There is a broad and growing base of research examining associations between prenatal exposure to non-persistent chemicals and fetal growth. For the sake of concision, this review highlighted results that met statistical significance at p<0.05. However, lack of statistically significance does not necessarily imply no true causal effect. We have not quantified results in any meta-analyses, nor attempted to evaluate to what extent the available literature may be influenced by publication bias. Our focus, rather, has been to discuss the strengths and limitations of the state of the epidemiologic literature of the associations between maternal prenatal biomarkers of non-persistent environmental chemicals and fetal growth. There are a number of factors – from measurement of biomarkers to outcome assessment to statistical design – that may have influenced the lack of coherent conclusions amongst the studies we identified. This literature is characterized by variability in exposure and outcome assessment, as well as analytical decision-making. Such variability likely contributes to the inconsistency of published results. To improve understanding of how everyday chemical exposures affect fetal growth, it is necessary to examine these questions with improved study designs and more consistency across analyses.

## Conclusions

The purpose of this review was to summarize the existing literature regarding biomarkers of prenatal non-persistent environmental chemicals exposure and fetal growth. We highlighted three chemical groups: phthalates, environmental phenols and other non-persistent consumer product chemicals, and non-persistent pesticides. There is growing evidence that prenatal maternal exposure to some high molecular weight phthalates is related to perturbations in fetal growth measured during pregnancy and infant size measured at birth. BPA was the most extensively studies environmental phenol in this literature base, but it was generally not associated with fetal growth. Among the few studies of dichlorophenols and fetal growth, there is some evidence that exposure to this group of phenols is related to reduced fetal growth in utero and reduced size at birth. Organophosphates remain the most widely studied non-persistent pesticide in this literature, despite reductions in use over the past two decades. Research indicates that associations between levels of dialkyl phosphates and infant size at birth differ by genetic factors, though conclusions from the studies reviewed vary somewhat. We identified no studies of maternal prenatal biomarkers of non-persistent pesticides and fetal growth measured during gestation by ultrasound.

The ultimate value in determining whether prenatal exposure to non-persistent chemicals affects fetal growth lies in understanding if, how, and when it is possible to reduce exposure and thus adverse outcomes. Exposure to these classes of chemicals may be reduced by both individual and regulatory action [93, 258]. This review highlights the need for future research in this area that examines fetal growth trajectories over the course of gestation, multiple measures of both exposure biomarkers and outcome measures in utero, modification by fetal sex, and multiple chemical exposures. Strengthening and harmonizing methodology will improve comparison between studies, evaluation of existing research, and ultimately aid in recommendations for regulatory and individual actions.

## Additional files

Additional file 1: Keywords for literature review. Word document of keywords used in literature review. (DOCX 64 kb)

Additional file 2: Supplemental tables. Word document of Supplemental Tables S1, S2, and S3. (DOCX 43 kb)

## Abbreviations

11ß-HSD2: 11ß-hydroxysteroid dehydrogenase 2
2,4,6-TCP: 2,4,6-trichlorophenol
2,4-D: 2,4-dichlorophenoxyacetic acid
2,4-DCP: 2,4-dichlorophenol
2,5-DCP: 2,5,-dichlorophenol
25OHD: 25-hydroxyvitamin D
3-PBA: 3-phenoxybenzoic acid
BP3: benzophenone-3
BPA: bisphenol A
DAPs: dialkyl phosphates
DBP: dibutyl phthalate
DEHP: di(2-ethylhexyl) phthalate
EDCs: endocrine disrupting compounds
ICC: intraclass correlation coefficient
IGF-2: insulin-like growth factor 2
ip-PPP: isopropyl-phenyl phenyl phosphate
IUGR: intrauterine growth restriction
MBzP: monobenzyl phthalate
MCNP: monocarboxy-isononyl phthalate
MEP: monoethyl phthalate
MMP: monomethyl phthalate
MnBP: mono-*n*-butyl phthalate
OPPs: organophosphate pesticides
PPARs: peroxisome proliferator activated receptors
PVC: polyvinyl chloride
SGA: small for gestational age
TCC: triclocarban
TCPy: 3,5,6-trichloro-2-pyridinol
TCS: triclosan
U.S.: United States
