# Supplementary material for: Fetal growth in environmental epidemiology: mechanisms, limitations, and a review of associations with biomarkers of non-persistent chemical exposures during pregnancy
Source: Environ Health. 2019 May 8;18:43. doi: 10.1186/s12940-019-0480-8 (PMC6505101; doi:10.1186/s12940-019-0480-8)
Supplement: Supplementary file 1 — Keywords for literature review. Word document of keywords used in literature review. (DOCX 64 kb) [file 12940_2019_480_MOESM1_ESM.docx]

Additional File 1. **Keywords for literature review**

Fetal growth keywords included: fetal weight, fetal growth, fetal overgrowth, biometric parameter, humerus length, femur length, fundal height, occipital frontal diameter, abdominal circumference, biparietal diameter, head circumference, crown rump length, gestational age, sga, lga, birth weight, birth length, intrauterine growth, large-for-date, small-for-date, macrosomia, overgrowth, growth restriction, growth retardation, iugr.

Non-persistent environmental exposure keywords included: non-persistent, phthalate, bisphenol, triclosan, phenol, chlorophenol, paraben, pesticide, organophosphate, diazinon, carbamate, carbaryl, chlorpyrifos, phosphate, malathion, parathion, fenchlorphos, trichlorophenol, fungicide, o-phenylphenol, OPP, herbicide, chlorophenoxy, triazine, atrazine, chloroacetamide, alachlor, pyrethroid, permethrin. repellant, deet, naphthol, dichlorophenol, nicotinoid, dicarboximide, insecticide.

We attempted to further refine results to relevant studies in human populations by using combinations of words including, but not limited to: cohort, review, meta-analysis, case-control, randomized, cross-sectional, survey, surveillance, women, fetus, fundus, foetus, infant, newborn, mother, twin, singleton.

Additionally, we examined the references sections of relevant studies to identify additional relevant research. More than 3000 results were produced using these search criteria.
