# Supplementary material for: Fetal growth in environmental epidemiology: mechanisms, limitations, and a review of associations with biomarkers of non-persistent chemical exposures during pregnancy
Source: Environ Health. 2019 May 8;18:43. doi: 10.1186/s12940-019-0480-8 (PMC6505101; doi:10.1186/s12940-019-0480-8)
Supplement: Supplementary file 2 — Supplemental tables. Word document of Supplemental Tables S1, S2, and S3. (DOCX 43 kb) [file 12940_2019_480_MOESM2_ESM.docx]

Supplemental Tables

Supplemental Table S1. Studies of maternal non-urinary phthalate biomarkers and fetal growth outcomes measured at birth.

| **Reference** | **Country, years** | **N** | **Phthalates measured** | **Growth outcome(s)** | **Notes^1^** | **All^2^** | **Girls^2^** | **Boys^2^** |
| --- | --- | --- | --- | --- | --- | --- | --- | --- |
| Latini et al. 2003 [118] | Italy  years unknown | 84 | DEHP, MEHP in cord serum | BW | not adjusted for GA | NS |  |  |
| Huang et al. 2009 [117] | Taiwan  2005-2006 | 65 | MMP, MEP, MBP, MBzP, MEHP in amniotic fluid | BW  BL | planning to undergo amniocentesis, not adjusted for GA |  | + BW (MBP)  + BL (MBP) | NS |
| Zhang et al. 2009 [120] | China  2005-2006 | 314 | DEP, DBP, DEHP, MEHP, MBP, MEHP in cord serum; MBP, MEHP in meconium | LBW | ≥37 weeks only | + LBW (cord serum DBP, MEHP; meconium MBP, MEHP) |  |  |
| Brucker-Davis et al. 2010 [114] | France  2002-2005 | 86 | DBP, MBP in cord serum | BW  BL  HC | ≥34 weeks only, not adjusted for GA |  |  | + HC (MBP) |
| Huang et al. 2014 [117] | China  2011-2012 | 207 | DMP, DEP, DIBP, DBP, DMEP, BMPP, DEEP, DPP, DNHP, BBP, DBEP, DCHP, DEHP, DNOP, DNP in cord blood` | BW  BL  AC  BPD  FL |  |  | - BW (DEEP)  - AC (DEP, DNHP, BBP, DNP)  - FL (DBP, DCHP, DEHP, DEP)  - HC (DNHP) | - BL (DPP, DBEP) |
| Xie et al. 2015 [123] | China  2011 | 185 | MBP, MEHP in meconium | BW  BL  LBW | ≥37 weeks only | - BW (MEHP, MBP)  - BL (MEHP, MBP)  + LBW (MEHP, MBP) |  |  |
| de Cock et al. 2016 [115] | The Netherlands  2011-2013 | 91 | MECPP, MEHHP, MEOHP in cord plasma | BW |  |  | NS | - BW (MECPP)  + BW (MEHHP) |
| Govarts et al. 2016 [116] | Belgium  2008-2009 | 248 | MECPP in cord plasma | BW |  | NS | NS | NS |
| Kim et al 2016 [125] | Korea  2011-2012 | 73 | ∑DEHP: MEHHP, MEOHP in newborns’ urine | BW  BL  HC |  | NS | NS | + BL (MEHHP, MEOHP, ∑DEHP) |

Supplemental Table S1 continued.

| **Reference** | **Country, years** | **N** | **Phthalates measured** | **Growth outcome(s)** | **Notes^1^** | **All^2^** | **Girls^2^** | **Boys^2^** |
| --- | --- | --- | --- | --- | --- | --- | --- | --- |
| Lenters et al. 2016 [121] | Greenland, Poland, and Ukraine  2002-2004 | 1250 | ∑DEHP: MEHHP, MEOHP, MECPP  ∑DiNP: MHiNP, MOiNP, MCiOP in maternal serum taken in early to mid-pregnancy | BW | ≥37 weeks only | - BW (MEHHP, MOEHP, ∑DEHP) | - BW (MEHHP) | NS |
| Li et al. 2016 [119] | China  years unknown | 187 | BBP, DEP, DEHP, DMP, DNOP in cord serum | BW  BL |  | + BW (BBP) | + BW (BBP, DNOP) | - BW (DEHP) |
| Minatoya et al. 2017 [122] | Japan  2002-2005 | 167 | MEHP in maternal serum at 34.3 weeks | BW | ≥37 weeks only | NS | NS | NS |

^1^Unless otherwise specified, studies included term, preterm, parous, and nulliparous births, and models of birth outcomes adjusted for or standardized to gestational age.

^2^Blank cells indicate associations were not examined; NS indicates that associations within that category were examined but not statistically significant at p<0.05.

Abbreviations. AC, abdominal circumference; BBP, benzyl butyl phthalate; BPD, biparietal diameter; BL, birth length; BMPP, bis (4-methyl-2-pentyl) phthalate; BW, birth weight; DBEP, di-2-nbutoxyethyl phthalate; DCHP, dicyclohexyl phthalate; DEEP, di-2-ethoxyethyl phthalate; DEHP, di-2-ethylhexyl phthalate; DBP, dibutyl phthalate; DEP, diethyl phthalate; DIBP, diisobutyl phthalate; DMEP, di-2-methoxyethyl phthalate; DMP, dimethyl phthalate; DNHP, dihexyl phthalate; DNOP, di-n-octyl phthalate; DNP, dinonyl phthalate; DPP, diamyl phthalate; FL, femur length; GA, gestational age; HC, head circumference; LBW, low birth weight; MBP, monobutyl phthalate; MBzP, monobenzyl phthalate; MCiOP, mono(4-methyl-7-carboxyheptyl) phthalate; MEP, monoethyl phthalate; MECPP, mono-2-ethyl-5-carboxypentyl phthalate; MEHHP, mono-2-ethyl-5-hydroxyhexyl phthalate; MEHP, mono-2-ethylhexyl phthalate; MEOHP, mono-2-ethyl-5-oxohexylphthalate; MHiNP, mono-hydroxy-iso-nonyl phthalate; MMP, monomethyl phthalate; MOiNP, mono-oxo-iso-nonyl phthalate; ∑DEHP, molar sum of the di-2-ethylhexyl phthalate metabolites that follow, ∑DiNP, molar sum of the di-iso-nonyl phthalate metabolites that follow.

Supplemental Table S2. Studies of maternal non-urinary biomarkers of phenols^1^ and fetal growth outcomes measured at birth.

| **Reference** | **Country, years** | **N** | **Phenol(s)** | **Outcome(s)** | **Notes^2^** | **All^3^** | **Girls^3^** | **Boys^3^** |
| --- | --- | --- | --- | --- | --- | --- | --- | --- |
| Padmanabhan et al. 2008 [165] | USA  2006 | 40 | BPA; maternal blood, plasma, and serum at delivery | BW |  | NS |  |  |
| Chou et al. 2011 [164] | Taiwan  2006-2007 | 97 | BPA; maternal plasma at delivery | LBW  SGA | ≥37 weeks only | - LBW (BPA)  - SGA (BPA) | - SGA (BPA) | - LBW (BPA)  - SGA (BPA) |
| Burstyn et al. 2013 [169] | Canada  2005-2007 | 550 | BPA; 15-16 weeks maternal serum | SGA | ≥37 weeks only | NS | NS | NS |
| Troisi et al. 2014 [166] | USA  2007-2010 | 200 | BPA; placenta | LBW  SGA  LGA |  | - LBW (BPA)  - SGA (BPA) |  |  |
| Veiga-Lopez et al. 2015 [167] | USA  years unknown | 80 | BPA in maternal plasma at 9-14 weeks and delivery and in cord blood | BW | not adjusted for GA | - BW (early pregnancy BPA) | - BW (early pregnancy BPA)  + BW (late pregnancy BPA) | NS |
| Xu et al. 2015 [168] | China  2010-2011 | 200 | BPA; cord serum | BW | not adjusted for GA | NS |  |  |
| Geer et al. 2016 [150] | USA  2007-2009 | 185 | BePB, BuPB, EtPB, MePB, PrPB, TCC, TCS; cord plasma | BW  BL  HC  LBW | not adjusted for GA | - BL (PrPB) |  |  |
| Pinney et al. 2017 [170] | USA  2004-2006 | 130 | BPA; amniotic fluid 17.2 weeks | BW | ≥37 weeks only | - BW (BPA) |  |  |
| Krause et al. 2018 [152] | Denmark  2012-2014 | 157 | 4-HBP, maternal serum 18 weeks | BW  BL  HC |  |  | NS | - BW (4-HBP)  - HC (4-HBP) |

^1^Includes environmental phenols and other non-persistent consumer product chemicals

^2^Unless otherwise specified, studies included term, preterm, parous, and nulliparous births, and models of birth outcomes adjusted for or standardized to gestational age.

^3^Blank cells indicate associations were not examined; NS indicates that associations within that category were examined but not statistically significant at p<0.05. Note that (-) associations for low birth weight (LBW) and small for gestational age (SGA) indicate *decreased risk* for these outcomes, while (+) associations indicated *increased risk* with increased exposure.

Abbreviations. 4-HBP, 4-hydroxy-benzophenone; BePB, benzyl paraben; BL, birth length; BPA, bisphenol-A; BuPB, butyl paraben; BW, birth weight; EtPB, ethyl paraben; GA, gestational age; HC, head circumference; LBW, low birth weight; MePB, methyl paraben; PrPB, propyl paraben; SGA, small for gestational age; TCC, triclocarban; TCS, triclosan

Supplemental Table S3. Non-urine biomarkers of non-persistent pesticides and fetal growth outcomes measured at birth

| **Reference** | **Country, years** | **N** | **Exposure(s)** |  | **Outcome(s)** | **Notes^1^** | **Results^2^** |
| --- | --- | --- | --- | --- | --- | --- | --- |
| Whyatt et al. 2005 [194] | USA  1998-2004 | 314 | 2-Isopropoxyphenol  ∑OP: Chlorpyrifos, diazinon, | Cord plasma | BW  BL  HC | born before 1/1/2001 | - BW (chlorpyrifos, ∑OP)  - BL (chlorpyrifos, ∑OP, 2-isopropoxyphenol) |
|  |  |  |  |  |  | born after 1/1/2001 | NS |
| Barr et al. 2010 [192] | USA  2003-2004 | 150 | Chlorpyrifos, diazinon, carbofuran, chlorothanlonil, dacthal, dichloran, metolachlor, trifluralin, DEET | cord serum, maternal serum at delivery | BW  BL  HC  AC | ≥37 weeks, elective cesarean section births only | + AC (dichloran in cord serum) |
| Neta et al. 2011 [193] | USA  2004-2005 | 185 | Permethrin: *cis*- and *trans*-permethrin isomers and PBUT | cord serum | BW  BL  HC |  | NS |
| Wickerham et al. 2012 [196] | China  2009 | 116 | Chlorpyrifos, diazinon, fonofos, malathion, parathion-ethyl, parathion-methyl, profenofos, terbufos; carbofuranphenol, propoxur, acetochlor, alachlor, atrazine, linuron, metolachlor, trifluralin, DEET  Fungicides: dichloran, metalaxyl, vinclozolin | cord serum | BW | ≥37 weeks only | - BW (number of pesticides detected, number of fungicides) |
| Koutroulakis et al. 2014 [197] | Greece  2006-2008 | 415 | ∑DMP: DMP, DMDTP, DMTP  ∑DEP: DEP, DEDTP, DETP  ∑DAP: DMP, DMDTP, DMTP, DEP, DEDTP, DETP | amniotic fluid at 16-20 weeks | BW  HC |  | + BW (∑DMP) |

^1^Unless otherwise specified, studies included term, preterm, parous, and nulliparous births, and models of birth outcomes adjusted for or standardized to gestational age.

^2^Blank cells indicate associations were not examined; NS indicates that associations within that category were examined but not statistically significant at p<0.05.

Abbreviations. AC, abdominal circumference; BL, birth length; BW, birth weight; DEET, diethyl-m-toluamide; DEP, diethylphosphate; DEDTP, diethyldithiophosphate; DETP, diethylthiophosphate; DMP, dimethylphosphate; DMDTP, dimethyldithiophosphate; DMTP, dimethylthiophosphate; HC, head circumference; ∑OP: Sum of chlorpyrifos and diazinon in chlorpyrifos equivalents; PBUT, piperonyl butoxide; ∑DAP, summed dialkyl phosphate metabolites; ∑DEP, summed diethyl phosphate metabolites; ∑DMP, summed dimethyl phosphate metabolites.
